# A phase transition induces chaos in a predator-prey ecosystem with a dynamic fitness landscape

**DOI:** 10.1371/journal.pcbi.1005644

**Published:** 2017-07-05

**Authors:** William Gilpin, Marcus W. Feldman

**Affiliations:** 1 Department of Applied Physics, Stanford University, Stanford, California, United States of America; 2 Department of Biology, Stanford University, Stanford, California, United States of America; University of California Irvine, UNITED STATES

## Abstract

In many ecosystems, natural selection can occur quickly enough to influence the population dynamics and thus future selection. This suggests the importance of extending classical population dynamics models to include such eco-evolutionary processes. Here, we describe a predator-prey model in which the prey population growth depends on a prey density-dependent fitness landscape. We show that this two-species ecosystem is capable of exhibiting chaos even in the absence of external environmental variation or noise, and that the onset of chaotic dynamics is the result of the fitness landscape reversibly alternating between epochs of stabilizing and disruptive selection. We draw an analogy between the fitness function and the free energy in statistical mechanics, allowing us to use the physical theory of first-order phase transitions to understand the onset of rapid cycling in the chaotic predator-prey dynamics. We use quantitative techniques to study the relevance of our model to observational studies of complex ecosystems, finding that the evolution-driven chaotic dynamics confer community stability at the “edge of chaos” while creating a wide distribution of opportunities for speciation during epochs of disruptive selection—a potential observable signature of chaotic eco-evolutionary dynamics in experimental studies.

## Author summary

Evolution is usually thought to occur very gradually, taking millennia or longer in order to appreciably affect a species' survival mechanisms. Conversely, demographic shifts due to predator invasion or environmental change can occur relatively quickly, creating abrupt and lasting effects on a species survival. However, recent studies of ecosystems ranging from the microbiome to oceanic predators have suggested that evolutionary and ecological processes can often occur over comparable timescales—necessitating that the two be addressed within a single, unified theoretical framework. Here, we show that when evolutionary effects are added to a minimal model of two competing species, the resulting ecosystem displays erratic and chaotic dynamics not typically observed in such systems. We then show that these chaotic dynamics arise from a subtle analogy between the evolutionary concept of fitness, and the concept of the free energy in thermodynamical systems. This analogy proves useful for understanding quantitatively how the concept of a changing fitness landscape can confer robustness to an ecosystem, as well as how unusual effects such as history-dependence can be important in complex real-world ecosystems. Our results predict a potential signature of a chaotic past in the distribution of timescales over which new species can emerge during the competitive dynamics, a potential waypoint for future experimental work in closed ecosystems with controlled fitness landscapes.

## Introduction

In many natural ecosystems, at least one constituent species evolves quickly enough relative to its population growth that the two effects become interdependent. This phenomenon can occur when selection forces are tied to such sudden environmental effects as algal blooms or flooding [1], or it can arise from more subtle, population-level effects such as overcrowding or resource depletion [2]. Analysis of such interactions within a unified theory of “eco-evolutionary dynamics” has been applied to a wide range of systems—from bacteria-phage interactions to bighorn sheep [3]—by describing population fluctuations in terms of the feedback between demographic change and natural selection [4].

The resulting theoretical models relate the fitness landscape (or fitness function) to population-level observables such as the population growth rate and the mean value of an adapting phenotypic trait (such as horn length, cell wall thickness, etc). The fitness landscape may have an arbitrarily complex topology, as it can depend on myriad factors ranging from environmental variability [5, 6], to inter- and intraspecific competition [7, 8], to resource depletion [9]. However, these complex landscapes can be broadly classified according to whether they result in *stabilizing* or *disruptive* selection. In the former, the landscape may possess a single, global maximum that causes the population of individuals to evolve towards a state in which most individuals have trait values at or near this maximum [10]. Conversely, in disruptive selection, the fitness landscape may contain multiple local maxima, in which case the population could have a wide distribution of trait values and occupy multiple distinct niches [11].

In eco-evolutionary models, the shape of the fitness landscape may itself depend on the population densities of the interacting species it describes. Specifically, the concept that the presence of competition can lead a single-peaked fitness landscape to spontaneously develop additional peaks originates in the context of “competitive speciation” first proposed by Rosenzweig [12]. This is formalized in genetic models in which sympatric speciation is driven by competitive pressures rather than geographic isolation [13]. Competition-induced disruptive selection has been observed in natural populations of stickleback fish [14], microbial communities [15], and fruit flies [16, 17].

Here, we model eco-evolutionary dynamics of a predator-prey system based on first-order “gradient dynamics” [10, 18], a class of models that explicitly define the fitness in terms of the population growth rate *r*, which is taken to depend only on the mean value of the trait across the entire population, $\bar{c}$ [19]. Despite this simplification, gradient dynamics models display rich behavior that can account for a wide range of effects observed in experimental systems—in particular, recent work by Cortez and colleagues has shown that these models can result in irregular cycles and dynamical bifurcations that depend on the standing genetic variation present in a population [20, 21].

In our model, gradient dynamics cause the prey fitness landscape to change as a result of predation, and we find that the resulting dynamical system exhibits chaotic dynamics. Chaos is only possible in systems in which three or more dependent dynamical variables vary in time [22], and previously it has been observed in predator-prey systems comprising three or more mutually interdependent species, or in which an external environmental variable (such as seasonal variation or generic noise) is included in the dynamics [23, 24]. Here we show that evolution of just one species in a two-species ecosystem is sufficient to drive the ecosystem into chaos. Moreover, we find that chaos is driven by a density-dependent change of the fitness landscape from a stabilizing to disruptive state, and that this transition has hysteretic behavior with mathematical properties that are strongly reminiscent of a first-order phase transition in a thermodynamical system. The resulting dynamics display intermittent properties typically associated with ecosystems poised at the “edge of chaos,” which we suggest has implications for the study of ecological stability and speciation.

## Model

Adapting the notation and formulation used by Cortez (2016) [21], we use a two-species competition model with an additional dynamical variable introduced to account for a prey trait on which natural selection may act. The most general fitness function for the prey, *r*, accounts for density-dependent selection on a prey trait *c*,

$$\begin{array}{c} r\left(x,y,\bar{c},c\right)\equiv G\left(x,c,\bar{c}\right)-D\left(c,\bar{c}\right)-f\left(x,y\right), \end{array}$$ (1)

where *x* = *x*(*t*) is the time-dependent prey density, *y* = *y*(*t*) is the time-dependent predator density, *c* is a trait value for an individual in the prey population, and $\bar{c}=\bar{c}\left(t\right)$ is the mean value of the trait across the entire prey population at time *t*. *r* comprises a density-dependent birth rate *G*, a density-independent death rate *D*, and a predator-prey interaction term *f*, which for simplicity is assumed to depend on neither *c* nor $\bar{c}$. Thus the trait under selection in our model is not an explicit predator avoidance trait such as camouflage, but rather an endogenous advancement (i.e., improved fecundity, faster development, or reduced mortality) that affects the prey’s ability to exploit resources in its environment, even in the absence of predation.

The continuous-time “gradient dynamics” model that we study interprets the fitness *r* as the growth rate of the prey: [19, 25]

$$\begin{array}{c} \dot{x}=x\;r\left(x,y,\bar{c},c\right){|}_{c\to \bar{c}} \end{array}$$ (2)

$$\begin{array}{c} \dot{y}=y\;(f\left(x,y\right)-\tilde{D}\left(y\right)) \end{array}$$ (3)

$$\begin{array}{c} \dot{\bar{c}}=V\frac{\partial r(x,y,\bar{c},c)}{\partial c}{|}_{c\to \bar{c}}. \end{array}$$ (4)

Eq (2) is evaluated with all individual trait values *c* set to the mean value $\bar{c}$ because the total prey population density is assumed to change based on the fitness function, which in turn depends on the population-averaged value of the prey trait $\bar{c}$ [21]. The timescale of the dynamics in $\bar{c}$ are set by *V*, which is interpreted as the additive genetic variance of the trait [10]. While Eq (2) depends only on the mean trait value $\bar{c}$, the full distribution of individual trait values *c* present in a real-world population may change over time as the relative frequencies of various phenotypes change. In principle, additional differential equations of the form of Eq (4) could be added to account for higher moments of the distribution of *c* across an ensemble of individuals, allowing the gradient dynamics model to be straightforwardly extended to model a trait’s full distribution rather than just the population mean. However, here we focus on the case where the prey density dynamics $\dot{x}$ depend only on the mean trait value to first order, and we do not include differential equations for higher-order moments of the prey trait value distribution.

**Fig 1:**
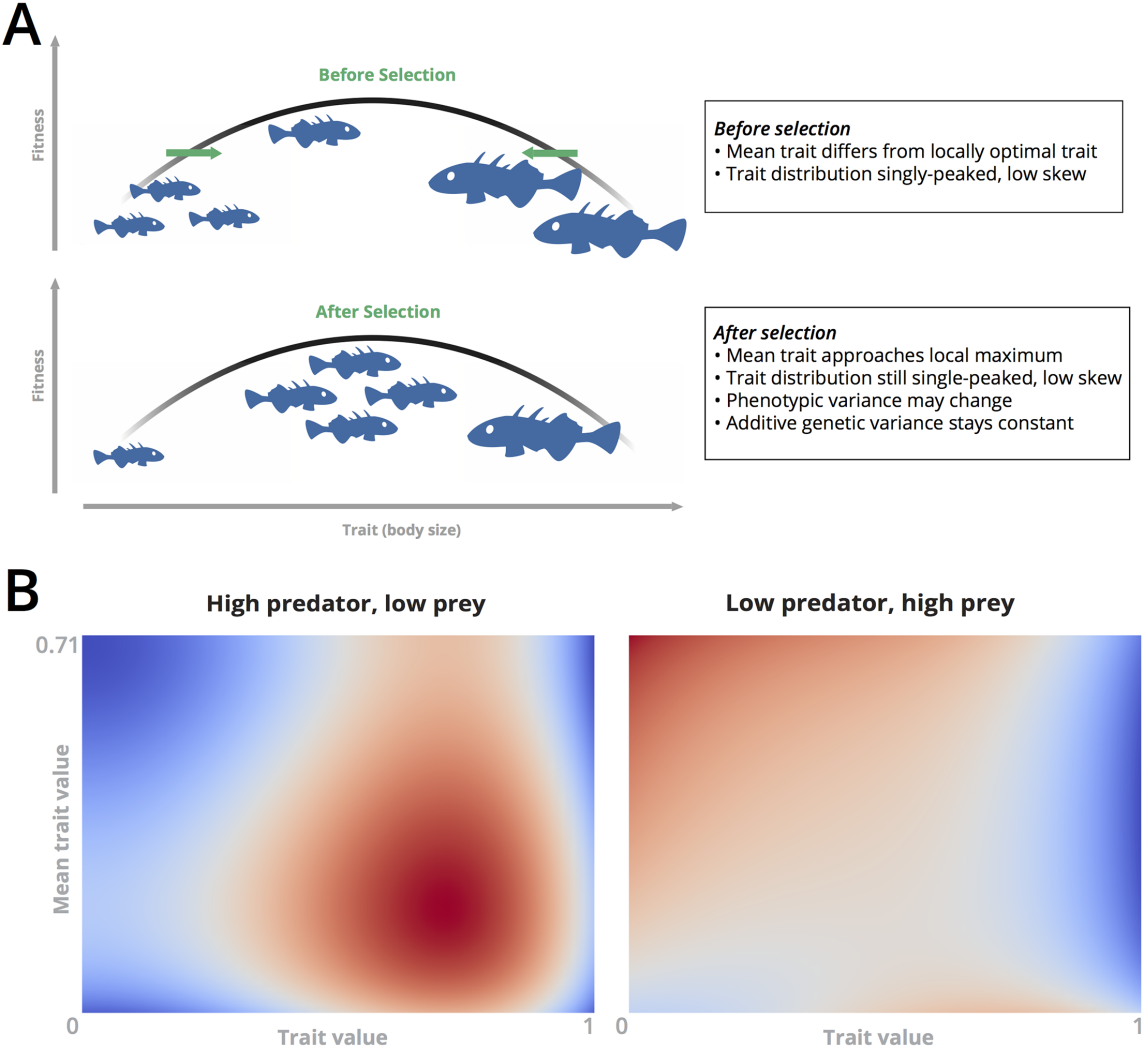
Description of model and fitness landscape. (A) A schematic depiction of the “gradient dynamics” model, illustrating the model’s assumptions regarding the role of selection in the evolutionary dynamics. (B) The two distinct topologies of the density-dependent fitness landscape, for typical values of the chosen parameters and observed predator and prey densities in the numerical work described below. Color scale ranges between −1 (blue) and 1 (red).

The use of a single Eq (4) to describe the full dynamics of the trait distribution represents an approximation that is exact only when the phenotypic trait distribution stays nearly symmetric and the prey population maintains a constant standing genetic variation *V* [10]. However, *V* may remain fixed even if the phenotypic variance changes, a property that is observed phenomenologically in experimental systems, and which may be explained by time-dependent heritability, breeding effects, mutation, or other transmission effects not explicitly modeled here [26–29]. More broadly, this assumption may imply that gene selection is weak compared to phenotype selection [30, 31]. S1D Appendix further describes the circumstances under which *V* remains fixed, and also provides a first-order estimate of the magnitude of error introduced by ignoring higher-order effects (such as skewness) in the trait distribution. The results suggest that these effects are small for the parameter values (and resulting range of *x* and *y* values) used here, due in part to limitations on the maximum skewness that a hypothetical trait distribution can achieve on the fitness landscapes studied here. In S1D Appendix, we also compare the results presented below to an equivalent model in which a full trait distribution is present, in which case Eq (2) becomes a full integro-differential equation involving averages of the trait value over the entire prey population. Detailed numerical study of this integro-differential equation is computationally prohibitive for the long timescales studied here, but direct comparison of the contributions of various terms in the velocity field suggests general accuracy of the gradient dynamics model for the fitness landscapes and conditions we study here. However, in general the appropriateness of the gradient dynamics model should be checked whenever using Eq (4) with an arbitrary fitness function. Fig 1A shows a schematic summarizing the gradient dynamics model, and noting the primary assumptions underlying this formulation.

Next, we choose functional forms for *f*, *G*, *D*, and $\tilde{D}$ in Eqs (2) and (3). We start with the assumption that, for fixed values of the trait *c* an d its mean $\bar{c}$, the population dynamics should have the form of a typical predator-prey system in the absence of evolutionary effects. Because the predator dynamics are not directly affected by evolutionary dynamics, we choose a simple form for predator growth consisting of a fixed death rate and a standard Holling Type II birth rate, [32]

$$\begin{array}{c} f\left(x,y\right)=a_{2}\frac{xy}{1+b_{2}x} \end{array}$$ (5)

$$\begin{array}{cc} \tilde{D}\left(y\right) & =d_{2} \end{array}$$ (6)

The predator birth rate *f* saturates at large values of the prey density, which is more realistic than the standard Lotka-Volterra competition term *xy* in cases where the prey density is large or fluctuating [22]. A saturating interaction term ensures that solutions of the system remain bounded for a wider range of parameter values, a necessity for realistic models of long-term interactions [33].

For the prey net growth rate (Eq (1), the fitness) in the absence of the predator, we use the following functional forms,

$$\begin{array}{c} G\left(x,\bar{c},c\right)=a_{1}\frac{\bar{c}}{1+b_{1}\bar{c}}(1-k_{1}x\left(c-\bar{c}\right)) \end{array}$$ (7)

$$\begin{array}{c} D\left(c,\bar{c}\right)=d_{1}\left(1-k_{2}\left(c^{2}-{\bar{c}}^{2}\right)+k_{4}\left(c^{4}-{\bar{c}}^{4}\right)\right). \end{array}$$ (8)

The first term in Eq (7) specifies that the prey population density growth rate ${r|}_{c\to \bar{c}}$ depends only on a primary saturating contribution of the mean trait to the birth rate *G*. In other models a similar effect is achieved by modifying the mean trait evolution Eq (4), such that extremal values of the trait are disadvantaged [21]; alternative coupling methods based on exponential saturation would be expected to yield similar qualitative results [19]. However, the additional series terms in Eqs (7) and (8) ensure that the any individual’s fitness *r* may differ from the rest of the population depending on the difference between its trait value *c* and the population mean $\bar{c}$. Because the functional form of this difference is unknown, its contribution expressed as second-order truncation of the series difference of the form $r\left(c,\bar{c}\right)=\tilde{r}{|}_{c\to 0}+\left(\tilde{r}\left(c\right)-\tilde{r}\left(c\right){|}_{c\to \bar{c}}\right)$ (where $\tilde{r}$ represents an unscaled fitness function). This ensures that when $\dot{c}=0$ or $c=\bar{c}$, the system reduces to a standard prey model with a Holling Type II increase in birth rate in response to increasing mean trait value [25]. In the results reported below, we observe that all dynamical variables remain bounded as long as parameter values are chosen such that the predator density does not equilibrate to zero. This is a direct consequence of our use of saturating Holling Type II functional forms in Eqs (7) and (8), which prevent the fitness landscape from increasing without bound at large *c*, $\bar{c}$ and also ensure that the predator and prey densities do not jointly diverge. That the dynamics should stay bounded due to saturating terms is justified by empirical studies of predator-prey systems [34, 35]; moreover, other saturating functional forms are expected to yield similar results if equivalent parameter values are chosen [33, 36].

The nonlinear dependence of the mortality rate Eq (8) on the trait is based on mechanistic models of mortality with individual variation [19, 37, 38]. The specific choice of a quartic in Eq (8) allows the fitness function *r* to have a varying number of real roots and local maxima in the domain *c*, $\bar{c}>0$, affording the system dynamical freedom not typically possible in predator prey models with constant or linear prey fitness—in particular, for different values of *k*_2_, *k*_4_ the fitness landscape can have a single optimal phenotype, multiple optimal phenotypes, or no optimal intermediate values. Because any even, continuous form for the fitness landscape can be approximated using a finite number of terms in its Taylor series around *c* = 0, our choice of a quartic form simply constitutes truncation of this expansion at the quartic order in order to include the simplest case in which the fitness function admits multiple local maxima—for this reason, a quartic will always represent the leading-order series expansion of a fitness landscape with multiple local maxima. Below, we observe numerically that $|c-\bar{c}|<1$, *ex post facto* justifying truncation of the higher order terms in this series expansion. However, if the trait value *c* was strictly bounded to only take non-zero values on a finite interval (as opposed to the entire real line), then a second-order, quadratic fitness landscape would be sufficient to admit multiple local maxima (at the edges of the interval) [14]. However, the choice here of an unbounded trait value *c* avoids creating boundary effects, and it has little consequence due to the steep decay of the quartic function at large values of |*c*|, which effectively confines the possible values of $\bar{c}$ accessible by the system. In physics, similar reasons—unbounded domains, multiple local optima, and continuity—typically justify the use of quartic free energy functions in minimal models of systems exhibiting multiple energetic optima, such as the Ginzberg-Landau free energy used in models of superconducting phase transitions [39].

We note that the birth rate Eq (7) contributes a density-dependent term to the fitness function even in the absence of predation (*y* = 0) [21]. Unlike the death rate function, the effect of the individual trait value on this term is directional: the sign of $c-\bar{c}$ determines whether birth rates increase or decrease. As the population density *x* increases, the effect of these directional effects is amplified, consistent with the observed effect of intraspecific competition and crowding in experimental studies of evolution [40, 41]. The chaotic dynamics reported below arise from this density-dependent term because the term prevents the Jacobian of the system (2), (3) and (4) from having a row and column with all zeros away from the diagonal; in this case, the prey trait (and thus evolutionary dynamics) would be uncoupled from the rest of the system, and would thus relax to a stable equilibrium (as is necessary for a first-order single-variable equation). In that case, $\bar{c}$ would essentially remain fixed and the predator-prey dynamics would become two-dimensional in *x* and *y*, precluding chaos. For similar reasons, density-dependent selection has been found to be necessary for chaos in some discrete-time evolutionary models, for which chaotic dynamics require a certain minimum degree of association between the fitness and the trait frequencies [42].

Inserting Eqs (5), (7) and (8), into Eq (1) results in a final fitness function of the form

$$\begin{array}{c} r\left(x,y,\bar{c},c\right)=a_{1}\frac{\bar{c}}{1+b_{1}\bar{c}}(1-k_{1}x\left(c-\bar{c}\right))-d_{1}\;(1-k_{2}\left(c^{2}-{\bar{c}}^{2}\right)+k_{4}\left(c^{4}-{\bar{c}}^{4}\right))-a_{2}\frac{xy}{1+b_{2}x}. \end{array}$$ (9)

This fitness landscape is shown in Fig 1B, for typical parameter values and predator and prey densities used in the numerical results below. Depending on the current predator and prey densities, the local maximum of the system can appear in two different locations, which directly affects the dynamics described in the next section.

Inserting Eq (9) into Eqs (2), (3) and (4) results in a final form for the dynamical equations,

$$\begin{array}{c} \dot{x}=x\;(a_{1}\frac{\bar{c}}{1+b_{1}\bar{c}}-a_{2}\frac{y}{1+b_{2}x}-d_{1}) \end{array}$$ (10)

$$\begin{array}{c} \dot{y}=y\;(y_{a}a_{2}\frac{x}{1+b_{2}x}-d_{2}) \end{array}$$ (11)

$$\begin{array}{c} \dot{\bar{c}}=\bar{c}\;V\;(\left(2k_{2}d_{1}\right)-\left(4k_{4}d_{1}\right){\bar{c}}^{2}-\left(a_{1}k_{1}\right)\frac{x}{1+b_{1}\bar{c}}). \end{array}$$ (12)

Due to the Holling coupling terms, the form of these equations qualitatively resembles models of vertical, tritrophic food webs—the mean trait value $\bar{c}$ affects the growth rate of the prey, which in turn affects the growth rate of the predator [24, 32, 43]. The coupling parameter *y*_*a*_ introduces asymmetry into the competition when *y*_*a*_ ≠ 1; however, it essentially acts as a scale factor that only affects the amplitude of the *y* cycles and equilibria rather than the dynamics. Additionally, because the predator-prey interaction term Eq (5) is unaffected by the trait, our model contains no triple-product *x* *y* $\bar{c}$ interaction terms, which typically stabilize the dynamics.

## Results

### Dynamical analysis and observation of fitness landscape switching

For our analysis of the system (10), (11) and (12), we first consider the case where evolution proceeds very slowly relative to population dynamics. In the case of both no evolution (*V* = 0) and no predation (*y* = 0), the prey growth Eq (10) advances along the one-dimensional nullcline $\dot{y}$, $\dot{\bar{c}}=0$, *y* = 0. Depending on whether the fixed mean trait value $\bar{c}$ exceeds a critical value (${\bar{c}}^{†}\equiv d_{1}/\left(a_{1}-b_{1}d_{1}\right)$), the prey density will either grow exponentially ($\bar{c}>{\bar{c}}^{†}$) or collapse exponentially ($\bar{c}<{\bar{c}}^{†}$) because the constant $\bar{c}$ remains too low to sustain the prey population in the absence of evolutionary adaptation. The requirement that $\bar{c}>{\bar{c}}^{†}$ carries over to the case where a predator is added to the system but evolutionary dynamics remain fixed, corresponding to a two dimensional system advancing along the two-dimensional nullcline $\dot{\bar{c}}=0$. In this case, as long as $\bar{c}>{\bar{c}}^{†}$, the prey density can exhibit continuous growth or cycling depending in the relative magnitudes of the various parameters in Eqs (10) and (11). The appearance and disappearance of these cycles is determined by a series of bifurcations that depends on the values of $\bar{c}$ and *b*_1_, *b*_2_ relative to the remaining parameters *a*_1_, *a*_2_, *d*_1_, *d*_2_ (S1A Appendix).

**Fig 2:**
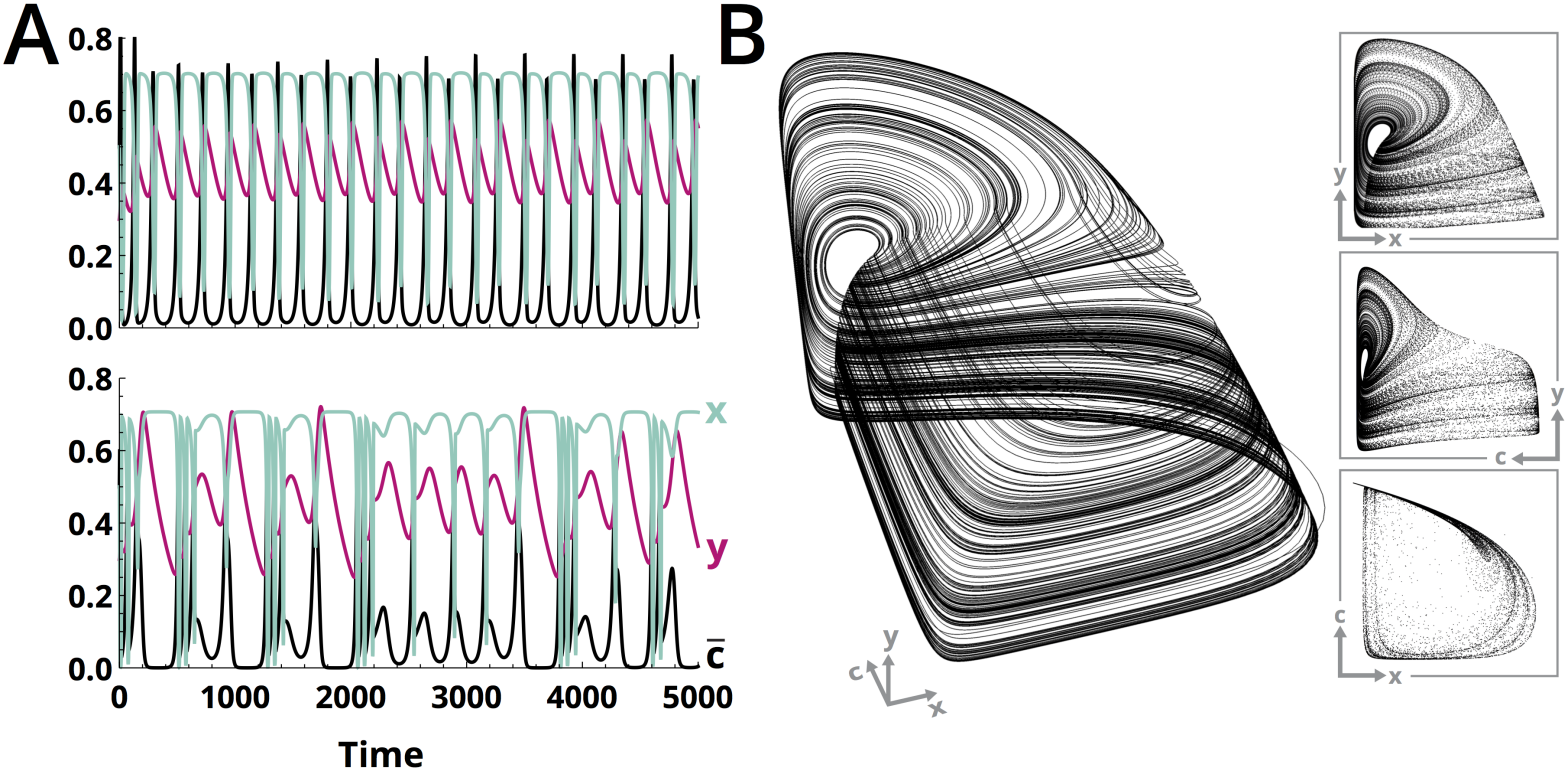
The onset of chaos with increasing prey evolutionary rate in a predator-prey system. (A) As the timescale of the evolutionary dynamics, *V*, increases relative to the timescales of predator-prey interactions, the system undergoes a Hopf bifurcation from stable limit cycles (upper panel, $V=0.\bar{06}$) to chaotic cycling (bottom panel, $V=0.\bar{3}$). (B) The “teacup” strange attractor for the system in its chaotic state ($V=0.\bar{3}$). Projections of the dynamics onto pairs of dynamical variables are shown in the inset. For this figure, *a*_1_ = 2.5, *a*_2_ = 0.05, *d*_1_ = 0.16, *d*_2_ = 0.004, *b*_1_ = 6, $b_{2}=1.\bar{3}$, *k*_1_ = 6, *k*_2_ = 9, *k*_4_ = 9, *y*_*a*_ = 8, Simulation time: 4 × 10^4^.

In the full three-variable system (10), (11) and (12), $\bar{c}$ passes through a range of values as time progresses, resulting in more complex dynamics than those observed in the two-dimensional case. For very small values of *V*, the evolutionary dynamics $\dot{\bar{c}}$ are slow enough that the system approaches the equilibrium predicted by the two-variable model with $\bar{c}$ constant. The predator and prey densities initially grow, but the prey trait value does not change fast enough for the prey population growth to sustain—eventually resulting in extinction of both the predator and prey. However, if *V* takes a slightly larger value, so that the mean trait value can gradually change with a growing prey population density (due to the density-dependent term in Eq (10)), then the population dynamics begin to display regular cycling with fixed frequencies and amplitudes (Fig 2A, top). This corresponds to a case where the evolutionary dynamics are slow compared to the ecological dynamics, but not so slow as to be completely negligible. Finally, when *V* is the same order of magnitude as the parameters governing the ecological dynamics, the irregular cycles become fully chaotic, with both amplitudes and frequencies that vary widely over even relatively short time intervals (Fig 2A, bottom). Typically, the large *V* case would correspond to circumstances in which the prey population develops a large standing genetic variation [10, 44].

That the dynamics are chaotic, rather than quasi-periodic, is suggested by the presence of multiple broad, unevenly-spaced peaks in the power spectrum [45] (Figure A in S1E Appendix), as well as by numerical tabulation of the Lyapunov spectrum (described further below). Due to the hierarchical coupling of Eqs (10), (11) and (12), when plotted in three-dimensions the chaotic dynamics settle onto a strange attractor that resembles the “teacup” attractor found in models of tritrophic food webs [24, 46] (Fig 2B). Poincare sections though various planes of the teacup all appear linear, suggesting that the strange attractor is effectively two-dimensional—consistent with pairings of timescales associated with different dynamical variables at different points in the process (Figure B in S1E Appendix). In the “rim” of the teacup, the predator density changes slowly relative to the prey density and mean trait value. This is visible in a projection of the attractor into the $x-\bar{c}$ plane (Fig 2B, bottom inset). However, in the “handle” of the teacup, the mean trait value varies slowly relative to the ecological dynamics ($\dot{\bar{c}}\approx 0$), resulting in dynamics that qualitatively resemble the two-dimensional “reduced” system described above for various fixed values of $\bar{c}$ (Fig 2B, top inset).

**Fig 3:**
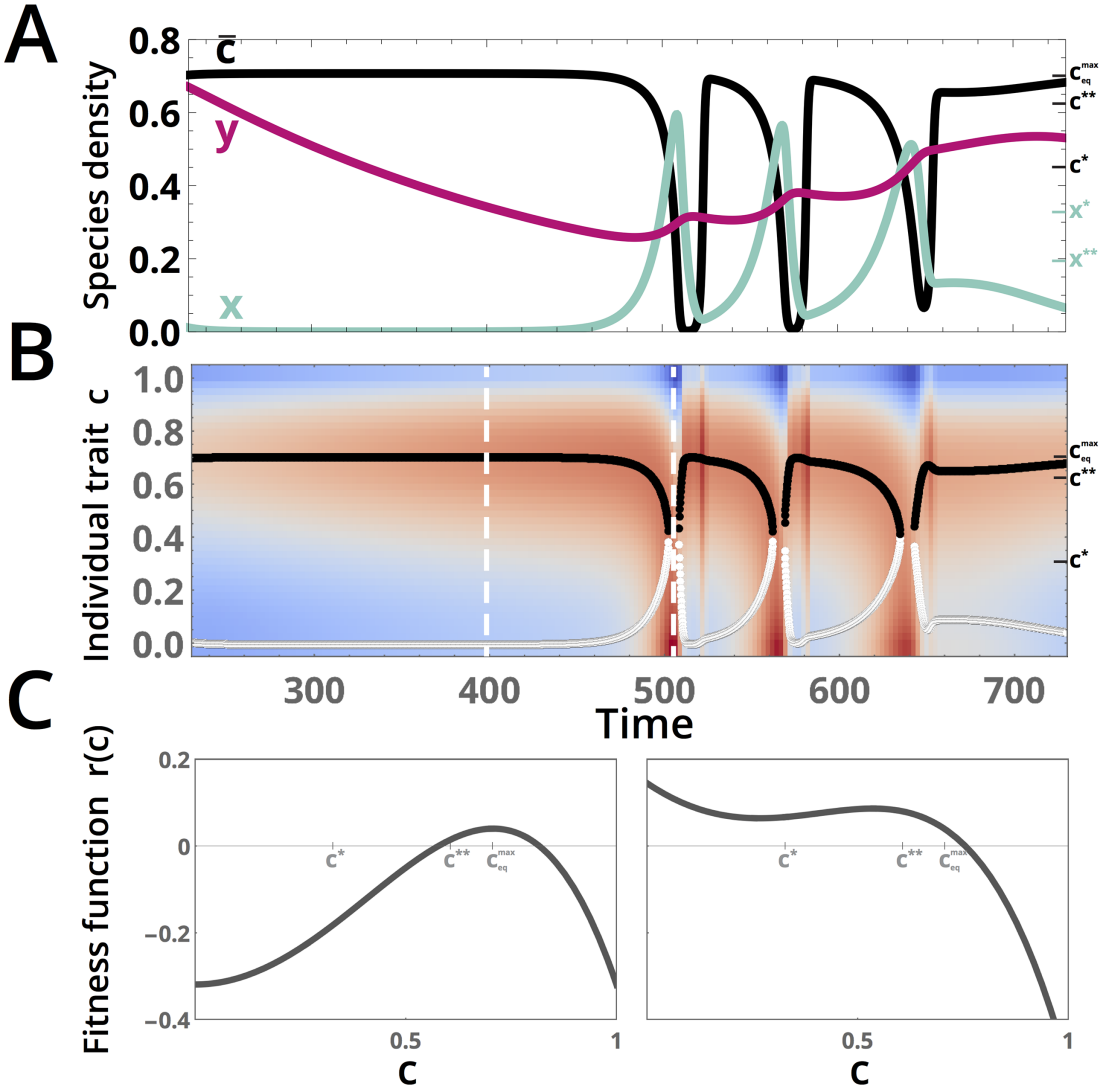
A gradual decrease in predator density remodels the fitness landscape, triggering transient cycling. (A) A closeup of the dynamics for a typical entry into cycling. A high predator density *y* (magenta) slowly decays while the prey density *x* (turquoise) remains nearly constant. Once the predator density becomes small enough, the prey density abruptly increases, causing a decrease in the mean trait value $\bar{c}$ (black) that provokes cycling. The right vertical axis ticks (x*, c*, etc.) correspond to analytical predictions for critical points, as described in the next section. (B) The fitness function *r* (from Eq (9)) computed for the values of (*x*, *y*, $\bar{c}$) at the timepoints shown in (A), plotted with *c* as the vertical axis. Local minima (white) and maxima (black) in *c* are overlaid for the portion of the dynamics shown in (A). The color gradient is centered with white at 0 and the positive (red) and negative(blue) fitness values scaled by the log transform modulus. (C) The fitness function as a function of *c* at two representative timepoints (indicated by white dashed lines in (B)). All parameters as given in Fig 2B.

The structure of the attractor suggests that the prey alternately enters periods of evolutionary change and periods of competition with the predator. A closer inspection of a typical transition reveals that this “two timescale” dynamical separation is responsible for the appearance of chaos in the system (Fig 3A). As the system explores configuration space, it reaches a metastable configuration corresponding to a high mean trait value $\bar{c}$, which causes the prey density to nearly equilibrate to a low density due to the negative density-dependent term in Eq (10). During this period (the “rim” of the teacup), the predator density gradually declines due to the lack of prey. However, once the predator density becomes sufficiently small, the prey population undergoes a sudden population increase, which triggers a period of rapid cycling in the system (the “handle” of the teacup attractor). During this time, the predator density continuously increases, causing an equivalent decrease in the prey density that resets the cycle to the metastable state.

The sudden increase in the prey population at low predator densities can be understood from how the fitness function *r* (from Eq (9)) changes over time. Fig 3B shows a kymograph of the log-scaled fitness Eq (9) as a function of individual trait values *c*, across each timepoint and corresponding set of (*x*, *y*, $\bar{c}$) values given in panel A. Overlaid on this time-dependent fitness landscape are curves indicating the instantaneous location of the local maximum (black) and minimum (white). By comparing panels A and B, it is apparent that the mean trait value during the “metastable” period of the dynamics stays near the local maximum of the fitness function, which barely varies as the predator density *y* changes. However, when *y*(*t*) ≈ 0.25, the fitness function changes so that the local minimum and local maximum merge and disappear from the system, leading to a new maximum spontaneously appearing at *c* = 0. Because *V* is large enough (for these parameters) that the gradient dynamics occur over timescales comparable to the competition dynamics, the system tends to move rapidly towards this new maximum in the fitness landscape, resulting in rapidly-changing dynamics in *x* and $\bar{c}$. Importantly, because of the symmetric coupling of the prey fitness landscape *r* to the prey density *x*, this rapid motion resets the fitness landscape so that the maximum once again occurs at the original value, resulting in a period of rapid cycling. The fitness landscape at two representative timepoints in the dynamics is shown in Fig 3C.

That the maxima in the fitness Function (9) suddenly change locations with continuous variation in *x*, *y* is a direct consequence of the use of a high-order (here, quartic) polynomial in *c* to describe the fitness landscape. The quartic represents the simplest analytic function that admits more than one local maxima in its domain, and the number of local maxima is governed by the relative signs of the coefficients of the $\left(c^{2}-{\bar{c}}^{2}\right)$ and $\left(c^{4}-{\bar{c}}^{4}\right)$ terms in Eq (9), which change when the system enters the rapid cycling portion of the chaotic dynamics at *t* = 500 in Fig 3A. This transition marks the mean prey trait switching from being drawn (via the gradient dynamics) to a single fitness peak at an intermediate value of the trait *c*_*eq*_ ≈ 0.707 to being drawn instead to one of two peaks: the existing peak, or a new peak at the origin. Thus the metastable period of the dynamics corresponds to a period of stabilizing selection: if the fitness landscape were frozen in time during this period, then an ensemble of prey would all evolve to a single intermediate trait value corresponding to the location of the global maximum. Conversely, if the fitness landscape were held fixed in the multipeaked form it develops during a period of rapid cycling, given sufficient time an ensemble of prey would evolve towards subpopulations with trait values at the location of each local fitness maximum—representing disruptive selection. That the fitness landscape does not remain fixed for extended durations in either a stabilizing or disruptive state—but rather switches between the two states due to the prey density-dependent term in Eq (9)— underlies the onset of chaotic cycling in the model. Density-dependent feedback similarly served to induce chaos in many early discrete-time ecosystem models [23]. However, the “two timescale” form of the chaotic dynamics and strange attractor here is a direct result of reversible transitions between stabilizing and disruptive selection.

If the assumptions underlying the gradient dynamics model do not strictly hold—if the additive genetic variance *V* slowly varies via an additional dynamical equation, or if the initial conditions are such that significant skewness would be expected to persist in the phenotypic distribution, then the chaotic dynamics studied here would be transient rather than indefinite. While the general stability analysis shown above (and in the S1 Appendix) would still hold, additional dynamical equations for *V* or for high-order moments of the trait distribution would introduce additional constraints on the values of the parameters, which would (in general) increase the opportunities for the dynamics to become unstable and lead to diverging predator or prey densities. However, in some cases these additional effects may actually serve to stabilize the system against both chaos and divergence. For example, if additional series terms were included in Eq (8) such that the dependence of mortality rate on $\bar{c}$ and *c* had an upper asymptote [25], then $\dot{\bar{c}}=0$ would be true for a larger range of parameter values—resulting in the dynamical system remaining planar for a larger range of initial conditions and parameter values, precluding chaos.

### Relationship to first-order phase transitions

The transition between stabilizing and disruptive selection that occurs when the system enters a period of chaotic cycling is strongly reminiscent of a first-order phase transition. Many physical systems can be described in terms of a free energy landscape, the negative gradient of which determines the forces acting on the system. Minima of the free energy landscape correspond to equilibrium points of the system, which the dynamical variables will approach with first-order dynamics in an overdamped limit.

When a physical system undergoes a phase transition—a qualitative change in its properties as a single “control” parameter, an externally-manipulable variable such as temperature, is smoothly varied—the transition can be understood in terms of how the control parameter changes the shape of the free energy landscape. The Landau free energy model represents the simplest mathematical framework for studying such phase transitions: a one-dimensional free energy landscape is defined as a function of the control parameter and an additional independent variable, the “order parameter,” a derived quantity (such as particle density or net magnetization) with respect to which the free energy can have local minima or maxima. In a first-order phase transition in the Landau model, as the control parameter monotonically changes the relative depth of a local minimum at the origin decreases, until a new local minimum spontaneously appears at a fixed nonzero value of the order parameter—resulting in dynamics that suddenly move towards the new minimum, creating discontinuities in thermodynamic properties of the system such as the entropy [47]. First-order phase transitions are universal physical models, which have been used to describe a broad range of processes spanning from superconductor breakdown [48] to primordial black hole formation in the early universe [49].

In the predator-prey model with prey evolution, the fitness function is analogous to the free energy, with the individual trait value *c* serving as the “order parameter” for the system. The control parameter for the transition is the prey density, *x*, which directly couples into the dynamics via the density-dependent term in Eq (7). Because the fitness consists of a linear combination of this term in Eq (7) and a quartic landscape Eq (8), the changing prey density “tilts” the landscape and provokes the appearance of the additional, disruptive peak visible in Fig 3C. The appearance and disappearance of local maxima as the system switches between stabilizing and disruptive selection is thus analogous to a first-order phase transition, with chaotic dynamics being a consequence of repeated increases and decreases of the control parameter *x* above and below the critical prey densities x*, x** at which the phase transition occurs. Similar chaotic dynamics emerge from repeated first-order phase transitions in networks of coupled oscillators, which may alternate between synchronized and incoherent states that resemble the “metastable” and “rapid cycling” portions of the predator-prey dynamics [50].

**Fig 4:**
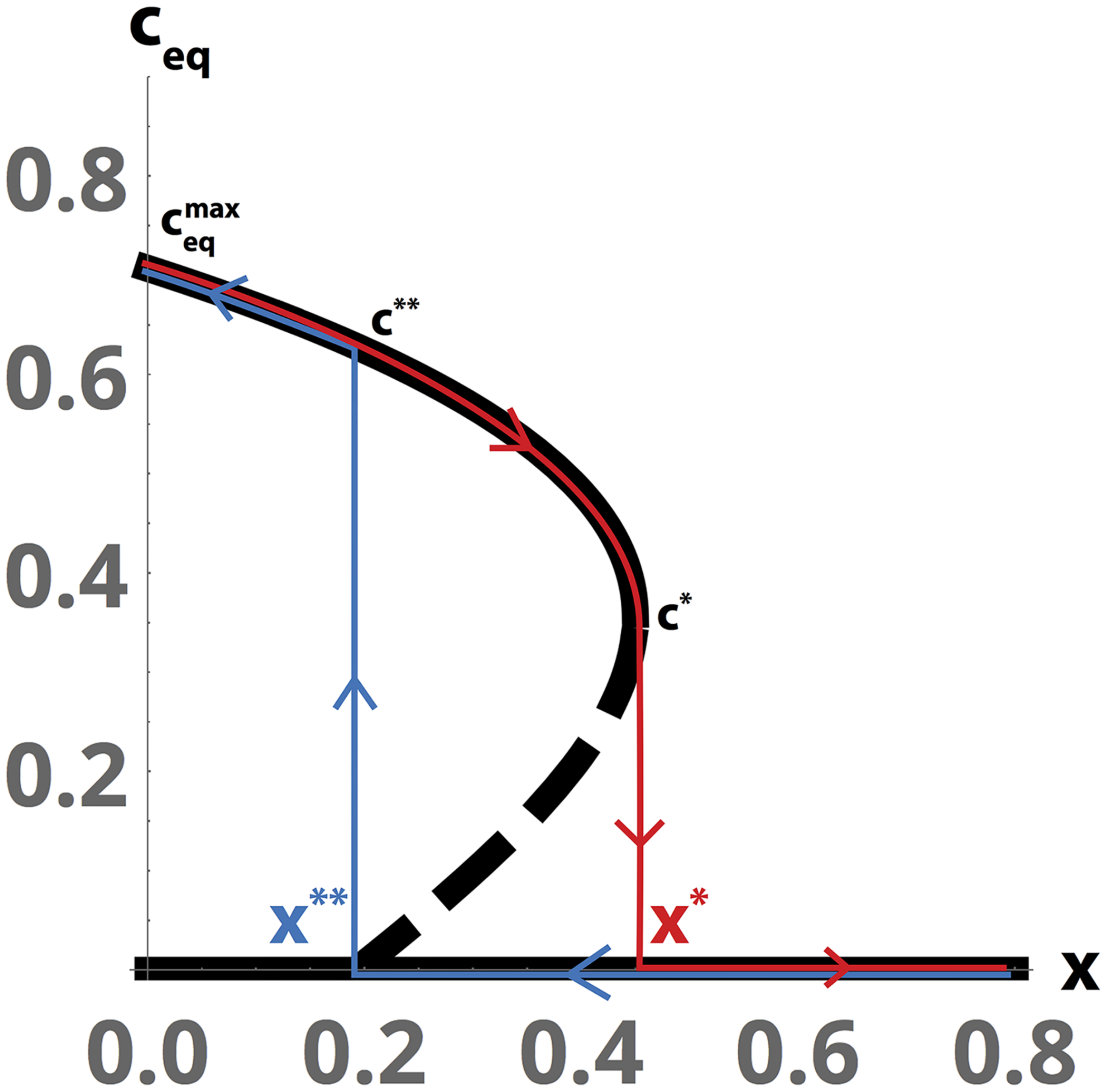
Hysteresis and discontinuity induced by a first-order fitness phase transition. (A) Under the fast equilibration approximation $\bar{c}\to c_{eq}$, an analytic phase diagram for the real parts of the locations of the local extrema of the fitness function as a function of the prey density, *x*. The red “forward” overlay indicates the apparent jump in the dynamics as *x* increases; the blue “backward” overlay indicates the apparent jump as *x* decreases. Dashed line indicates unstable equilibria. All parameters are as given in Fig 2B.

The analogy between a first-order phase transition and the onset of disruptive selection can be used to study the chaotic dynamics in terms of dynamical hysteresis, a defining feature of such phase transitions [47]. For different values of *x*, the three equilibria corresponding to the locations of the local minima and maxima of the fitness landscape, *c*_*eq*_, can be calculated from the roots of the cubic in Eq (12). The resulting plots of *c*_*eq*_ vs *x* in Fig 4 are generated by solving for the roots in the limit of fast prey equilibration, $\bar{c}\to c_{eq}$, which holds in the vicinity of the equilibria (S1B Appendix).

The entry into the transient chaotic cycling occurs when *x* increases gradually and shifts *c*_*eq*_ with it; *x* eventually attains a critical value x* (x* ≈ 0.45 for the parameters used in the figures), causing *c*_*eq*_ to jump from its first critical value c* to the origin (the red “forward” branch in Fig 4). This jump causes rapid re-equilibration of $\bar{c}\left(t\right)$, resulting in the rapid entry into cycling observable in Fig 3A. However, *x* cannot increase indefinitely due to predation; rather, it decreases until it reaches a second critical value x**, at which point *c*_*eq*_ jumps back from the origin to a positive value (the blue “return” branch in Fig 4; x** = 0.192 for these parameter values). This second critical point marks the return to the metastable dynamics in Fig 3A. This asymmetry in the forward and backwards dynamics of *x* lead to dynamical time-irreversibility (hysteresis) and the jagged, sawtooth-like cycles visible in the dynamics of the full system. Because the second jump in *c*_*eq*_ is steeper, the parts of the trajectories associated with the “return” transition in Fig 3A appear steeper. Additionally, the maximum value obtained by $\bar{c}\left(t\right)$ anywhere on the attractor, $c_{eq}^{max}$, is determined by the limiting value of *c*_*eq*_ as *x* → 0. Analytic values for $c_{eq}^{max}$, as well as (x*, c*) and (x**, c**), are derived in the S1 Appendix, and their corresponding numerical values are overlaid in each panel of Fig 3.

Comparing the values of $c_{eq}^{max}$, x*, c*, x**, c** to the dynamics of the system in Fig 3, it is apparent that calculation of critical points under the fast-evolution approximation correctly predicts key properties of the chaotic dynamics such as the maximum value attained by $\bar{c}\left(t\right)$, the quasi-static value $\bar{c}$ during the “metastable” period before chaotic cycling, and the approximate values at which *x*(*t*) enters and exits the rapid cycling portion of the dynamics. Thus the analogy between the fitness function and the Landau free energy provides insight into the dynamics of the chaotic ecosystem.

Moreover, for intermediate values of the prey density at which the two local maxima are equal heights, the relative fitnesses of the two trait values are equal ($c^{*}=c_{eq}^{**}$) and so both phenotypes would be equally favorable for the prey population. This is analogous to the coexistence of two phases during intermediate portions of a phase transition. As the prey density *x* approaches either critical value, the fitness landscape shallows and the dynamics begin to exhibit a form of “critical slowing down” associated with the onset of the phase transition—here represented by the relatively slow dynamics along the flattened handle of the teacup in Fig 2B.

### Implications for models of speciation

The chaotic dynamics reported here are emergent; they result from predation reducing the fitness of intermediate trait values, which restructures the fitness landscape in a manner that later reverses as the predator density decreases. However, here, as in other models, the presence of chaos has other long-term implications for the ecosystem that would not be relevant in systems with only limit cycles or point equilibria.

The chaotic dynamics associated with fast evolutionary dynamics (large *V*, or high genetic variance [20, 21]) impose a statistical structure on the deterministic problem: given a sufficiently long observation time, a trajectory along the strange attractor will sample every point on the attractor [45]. For the predator-prey model studied here, ergodicity in the system is established by using a numerical scheme to estimate the spectrum of global Lyapunov exponents, which measure the rate at which two infinitesimally separated points in the configuration space move apart over time along the three dimensions present in the system. Simulations with varying timescales that start at various initial conditions on the attractor converge to the same estimates of the Lyapunov exponents, implying ergodicity [45] (S1C Appendix). A similar technique has been used to establish ergodicity in some models of chaotic multitropic food webs [51]. The Lyapunov spectrum can, in turn, be used to determine the Kaplan-Yorke fractal dimension of the attractor, *D*_*KY*_ ≈ 2.01, which accounts for the two-dimensional shape of the full attractor (Fig 2B) and linear shapes of its Poincare sections (Figure B in S1E Appendix) discussed above.

Due to the ergodic property of chaotic attractors, one typical interpretation of their appearance in ecological dynamics is that they allow a sort of bet-hedging across timescales, conferring ecological stability against sudden external perturbations [23, 52, 53]. In the presence of external factors not explicitly included in the model, especially non-ergodic processes such as climate variation, a chaotic ecosystem will present a variety of different ratios of predator and prey concentrations at different times, ensuring robustness through biodiversity [54–56]. Moreover, in spatially-extended models in which different subpopulations may simultaneously exist at different points in the chaotic attractor, the chaotic attractor can allow one subgroup to recover from a sudden environmental catastrophe or to expand its range to a new location when favorable conditions spontaneously arise. In general, chaotic dynamics may present an adaptive benefit by making ecological networks robust, for example by preventing sudden exclusion of a keystone species [57].

Here, we suggest that chaos produces an additional effect when it arises due to eco-evolutionary dynamics: it creates a broad distribution of “windows” of time during which sympatric speciation may occur. The dynamics imposed in the predator-prey model in Eqs 10–12 do not explicitly include speciation, which represents an irreversible process in which the prey bifurcates into multiple co-evolving types (hence changing the number of distinct dependent variables present in the dynamics). This would violate the underlying conditions of the gradient dynamics model by creating a bimodal prey density vs trait distribution with substantial skew. However, this process would typically occur during periods of disruptive selection, during which speciation could occur either through assortative mating or through spatial isolation of phenotypically homogeneous subpopulations.

**Fig 5:**
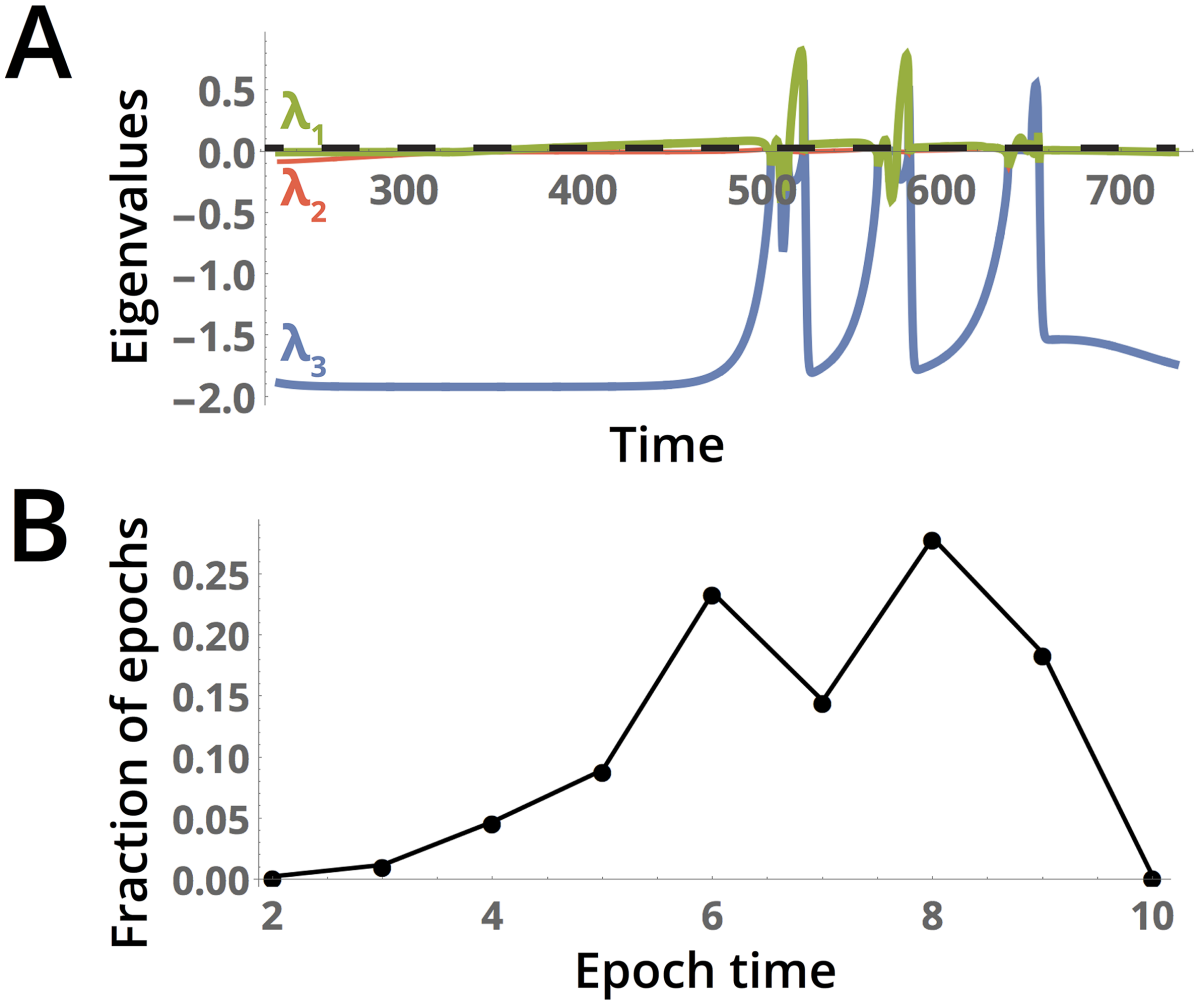
Lyapunov exponents associated with the chaotic dynamics. (A) The eigenvalues of the Jacobian evaluated during a portion of the dynamics in which the system enters a series of chaotic cycles. The global Lyapunov exponent is underlaid as a dashed black line. (B) The distribution of the lengths of periods in the dynamics during which the largest local Lyapunov exponent is greater than the threshold value 0.2, chosen to correspond to periods of strongly disruptive selection in which the exponent takes values an order of magnitude greater than its median across the time series. Simulation time 2 × 10^5^, other parameters are as given in Fig 2B.

For this reason, the statistical property of the chaotic dynamics that is relevant to speciation is the distribution of time that the fitness function spends in the disruptive state, or the “epochs” of disruptive selection. This represents the distribution of opportunities for speciation to first occur in the system, at which point ergodicity would be broken and the dynamical equations would no longer remain valid. In many models of evolutionary processes, the distribution of epochs of dominance for certain phenotypes has rich statistical structure, including a heavy-tail distribution that some authors have taken to indicate the presence of self-organized criticality [58, 59]. These epochs can be detected by defining the “local” Lyapunov exponents, which represent the three eigenvalues of the Jacobian matrix for the Systems (10), (11) and (12) evaluated at each point along a trajectory in the chaotic attractor [60, 61],

$$\lambda_{i}\left(t\right)\equiv \text{eig}\;(\frac{\partial \dot{x}\left(t\right)}{\partial x(t)})$$

where $x=(x\left(t\right),y\left(t\right),\bar{c}\left(t\right))$ and *i* ∈ {1, 2, 3}. Plots of these local Lyapunov exponents during a typical period of metastable dynamics followed by cycling are shown in Fig 5A. Positive values suggest chaotic dynamics, while negative values suggest that nearby trajectories converge. The largest local Lyapunov exponent typically dominates the dynamics. Consistent with the destabilizing nature of disruptive selection, the largest local Lyapunov exponent increases dramatically during periods in which the fitness function has multiple local maxima. For this reason, the length of these long excursions in which the largest local Lyapunov exponent significantly exceeds zero can be used to estimate the distribution lengths of periods of disruptive selection (Fig 5B), based on a very long sample of the dynamics along the strange attractor. The broadness of this distribution suggests that speciation events could occur over a range of timescales in the system (for example, via hybrid breakdown), representing a potential signature of a chaotic past that could be observed in descendant populations with non-chaotic dynamics.

Despite the large fluctuations in the maximum value of the local Lyapunov exponents, the largest global Lyapunov exponent is only barely larger than zero, *λ*_*max*_ ≈ 0.003. Similar behavior has been reported a real-world ecosystem consisting of competing species in a rocky intertidal environment, in which a small global Lyapunov exponent paired with a fluctuating largest local Lyapunov exponent was taken to suggest that the ecosystem had adapted to “the edge of chaos.” [51] A similar case has been reported experimentally in populations of voles in Northern Europe that appear to switch between chaotic and stable periods [62]. In that system, it was noted that occasional switches to chaotic dynamics serve to amplify the effect of environmental fluctuations, further suggesting that the irregular spacing of epochs resulting from chaotic dynamics may allow a range of timescales over which speciation may occur under temporally-varying external conditions.

If the underlying assumptions of the gradient dynamics model do not hold—such as *V* slowly varys in time or the trait distribution retains significant skewness—then the chaotic dynamics would be non-ergodic, causing the system to eventually exit the chaotic attractor and either diverge or settle to a fixed point or limit cycle. If the timescale of exit from the chaotic attractor is much longer than the average time between periods of rapid cycling (as determined, for example, by the peak in the power spectrum in Figure A of S1E Appendix), then the dynamics will demonstrate transient chaos, and the form of the distribution in Fig 5B will be roughly the same due to quasi-ergodicity. However, if the timescale of transience is much shorter, the dynamics may not fully sample the attractor, resulting in the distribution of epochs of disruptive selection being strongly dependent on the initial conditions.

## Discussion

We have shown that a simple two-species predator-prey ecosystem can display rich dynamical complexity when the prey evolves in response to predation, and that this complexity can be understood by analyzing the temporal variation of the fitness landscape. Future theoretical work will establish whether these dynamics qualitatively change when the predator also evolves over timescale comparable to the prey evolution [63]. Such predator-prey co-evolutionary systems have been shown to exhibit a distinct route to chaos, due a desynchronization of the predator and prey adaptation that comprises a form of the “Red Queen” effect [64, 65].

One limitation of our model arises from the form of the evolutionary dynamics Eq (4), which assume that the dynamics of the trait distribution can be adequately described by a mean trait evolution equation. S1D Appendix compares the results found here to those generated by a formulation of the problem in terms of a full integro-differential equation, and finds general agreement for the fitness landscape studied here. However, for more complicated fitness landscapes these conditions may not hold, requiring more advanced models that introduce additional dynamical equations to account for various effects such as non-constant additive genetic variance [30, 66, 67]. In such models, chaos may appear as a transient in the dynamics before the dynamical variables approach an equilibrium point or limit cycle.

Our findings for the minimal model studied here have implications for a wide variety of eco-evolutionary systems, because they suggest that even a minimal deterministic model can exhibit unstable cycling and chaos—effects that would typically become more pronounced when more species are added to the system [22, 23]. The mechanism by which chaos appears in our system is generic, resulting purely from changes in the number of local maxima in the fitness landscape, suggesting the applicability of our findings to observational systems (such as bacteria and viruses in microenvironments) in which the fitness landscape can be monitored, but not necessarily all of the underlying species interrelationships [68]. For these systems, recent advances in genetic barcoding of entire microbial communities [69, 70] may allow direct observation of the role of dynamic fitness landscapes in creating opportunities for sympatric speciation.

In addition to being an emergent property of the underlying species interactions, we suggest here that these chaotic properties may confer adaptive benefits via community robustness, either by enforcing phenotypic diversity or by preventing environmental variation from fully excluding a single species. The system described here also represents an example of a small ecosystem that adapts towards the “edge of chaos”, which can further adjust how the system responds to external perturbations [71, 72]. Potential experimental systems in which the adaptive role of eco-evolutionary chaos may be explored include phytoplanktonic ecosystems, which can be isolated in the laboratory and which are known to to maintain biodiversity using chaotic effects [54]. In particular, it would be interesting to determine whether non-synchronized replicates of experimentally-controlled chaotic ecosystems could recover from a synchronized perturbation (i.e. temporary salinity shock) more quickly than non-chaotic controls [74]—suggesting that the ability of chaotic systems to continuously sample a wide variety of dynamical conditions confers robustness. In these systems, reconstruction of of an experimental chaotic attractor derived from lagged coordinate embedding [43] could yield insight into whether chaos arises due to changes in the general topology of the fitness landscape, which would result in a nearly two-dimensional attractor due to distinct timescales associated with stabilizing and disruptive effects.

Moreover, the underlying cause of the chaotic dynamics—a reversible transition between stabilizing and disruptive selection—is mathematically analogous to the change in the shape of the free energy landscape during a first-order phase transition in thermodynamics. Our findings thus fit within more general extensions of mathematical theories of evolution that include formalism from statistical and condensed matter physics [8, 58, 72, 73], suggesting that universal mechanisms may underly subtle transient properties observed in many natural ecosystems, including hysteresis and dynamical robustness [75].

## Supporting information

S1 Appendix Detailed derivations of analytical results and additional numerical analysis. (PDF)
