# Supplementary material for: A phase transition induces chaos in a predator-prey ecosystem with a dynamic fitness landscape
Source: PLoS Comput Biol. 2017 Jul 5;13(7):e1005644. doi: 10.1371/journal.pcbi.1005644 (PMC5517034; doi:10.1371/journal.pcbi.1005644)
Supplement: S1 Appendix — (PDF) [file pcbi.1005644.s001.pdf]

Supplementary Material for "A phase transition induces chaos in a predator-prey ecosystem with a dynamic fitness landscape"

William Gilpin[1*], Marcus W. Feldman[2]

[1]Department of Applied Physics, [2]Department of Biology,

Stanford University, Stanford, CA

*wgilpin@stanford.edu

June 26, 2017

1 Supplementary Analysis

A Solutions in the absence of evolutionary dynamics

We study the system along the two-dimensional nullcline $\dot{\bar{c}} = 0$,

$$\dot{x} = x\left(a_1\frac{\bar{c}}{1+b_1\bar{c}} - a_2\frac{y}{1+b_2x} - d_1\right) \tag{A1}$$

$$\dot{y} = y\left(a_2\frac{y}{1+b_2x} - d\right), \tag{A2}$$

where $\bar{c}$ is a parameter representing some fixed mean trait value in the population. First, we consider the further reduced case of the prey density in the absence of predation, $\dot{y} = y = 0$. In this case, (A1) has the solution

$$x(t) = x_0 \exp\left(-\frac{(1+b_1\bar{c})d_1 - a_1\bar{c}}{1+b_1\bar{c}}t\right).$$

When $b_1d_1 < a_1$, the prey density grows exponentially if $\bar{c} > d_1/(a_1 - b_1d_1)$; it decreases exponentially when $\bar{c} < d_1/(a_1 - b_1d_1)$. Because a nonzero predator density only serves to decrease the rate of the prey growth, this constraint also applies to the two-dimensional case (A1, A2). These equations admit two solutions. The first is mutual exclusion,

$$\hat{x} = \hat{y} = 0, \tag{A3}$$

which has associated eigenvalues,

$$\lambda = -d_2 \tag{A4}$$

$$\lambda = \frac{a_1\bar{c} - d_1 - b_1d_1\bar{c}}{1+b_1\bar{c}}. \tag{A5}$$

As predicted for the one-dimensional problem, the mutual exclusion solution is stable when $\bar{c} < d_1/(a_1 - b_1d_1)$. However, even if $\bar{c} > d_1/(a_1 - b_1d_1)$, this solution is stable if competition

is strong enough that $b_1 > a_1/d_1$.

The second solution to (A1, A2) is an interior point,

$$\hat{x} = \frac{d_2}{a_2 - b_2 d_2} \tag{A6}$$

$$\hat{y} = -\frac{(-\bar{c}a_1 + \bar{c}b_1 d_1 + d_1)}{y_a(\bar{c}b_1 + 1)\,(a_2 - b_2 d_2)}. \tag{A7}$$

The associated eigenvalues for this solution occur in pairs,

$$\lambda_\pm = -\frac{1}{2(\bar{c}a_2 b_1 + a_2)}\Bigg(b_2 d_2(-\bar{c}a_1 + \bar{c}b_1 d_1 + d_1) \pm \sqrt{d_2(-\bar{c}a_1 + \bar{c}b_1 d_1 + d_1)(b_2^2 d_2(-\bar{c}a_1 + \bar{c}b_1 d_1 + d_1) + 4a_2^2(\bar{c}b_1 + 1) - 4a_2 b_2 d_2(\bar{c}b_1 + 1))}\Bigg). \tag{A8}$$

The requirement that $\hat{y}, \hat{x} \geq 0$ means that this interior solution exists only when $\bar{c} > d_1/(a_1 - b_1 d_1)$ (which, as before, entails exponential prey growth in the absence of the predator) and $b_1 < a_1/d_1, b_2 < a_2/d_2$. The latter two conditions are equivalent to the non-existence of coexistence solutions under strong competition in the classical Lotka-Volterra predator-prey model. For these conditions, the real parts of (A8) are always positive, and so the interior solution is never stable.

The eigenvalues of (A8) always have nonzero imaginary components if either

$$b_1 \geq \frac{a_1 d_2}{\frac{4a_2^2}{b_2^2} - \frac{4a_2 d_2}{b_2} + d_1 d_2},$$

or if

$$b_1 < \frac{a_1 d_2}{\frac{4a_2^2}{b_2^2} - \frac{4a_2 d_2}{b_2} + d_1 d_2},$$

$$\bar{c} < \frac{-4a_2^2 + 4a_2 b_2 d_2 - b_2^2 d_1 d_2}{-a_1 b_2^2 d_2 + 4a_2^2 b_1 - 4a_2 b_1 b_2 d_2 + b_1 b_2^2 d_1 d_2}.$$

When either of these conditions is satisfied, cycling is possible in the system.

B Hysteresis and critical points

Under the assumption that evolutionary dynamics are fast enough that $\bar{c} \approx c$ near the maximum of the fitness landscape $r(x, y, c, \bar{c})$, then c_{eq} are given by the solutions of the equation

$$\left(\frac{\partial r(x,y,c,\bar{c})}{\partial c} \bigg|_{c=c_{eq}} \right) \bigg|_{\bar{c}=c_{eq}} = 0.$$

The roots of this equations are intricate expressions; we define first the auxiliary variable β:

$$\beta \equiv 432 a_1 b_1^2 d_1^2 k_1 k_4^2 x - 576 b_1^2 d_1^3 k_2 k_4^2 + 128 d_1^3 k_4^3 \tag{A9}$$

$$+ \sqrt{\left(432 a_1 b_1^2 d_1^2 k_1 k_4^2 x - 576 b_1^2 d_1^3 k_2 k_4^2 + 128 d_1^3 k_4^3\right)^2 + 4\left(-24 b_1^2 d_1^2 k_2 k_4 - 16 d_1^2 k_4^2\right)^3}. \tag{A10}$$

The roots can now be written in the form,

$$c_{eq}^{(1)} = 0 \tag{A11}$$

$$c_{eq}^{(2)} = \frac{-24 b_1^2 d_1^2 k_2 k_4 - 16 d_1^2 k_4^2}{6\ 2^{2/3} b_1 \sqrt[3]{\beta} d_1 k_4} - \frac{\sqrt[3]{\beta}}{12 \sqrt[3]{2} b_1 d_1 k_4} - \frac{1}{3 b_1} \tag{A12}$$

$$c_{eq}^{(3)} = -\frac{\left(1 + i\sqrt{3}\right)\left(-24 b_1^2 d_1^2 k_2 k_4 - 16 d_1^2 k_4^2\right)}{12\ 2^{2/3} b_1 \sqrt[3]{\beta} d_1 k_4} + \frac{\left(1 - i\sqrt{3}\right)\sqrt[3]{\beta}}{24 \sqrt[3]{2} b_1 d_1 k_4} - \frac{1}{3 b_1} \tag{A13}$$

$$c_{eq}^{(4)} = -\frac{\left(1 - i\sqrt{3}\right)\left(-24 b_1^2 d_1^2 k_2 k_4 - 16 d_1^2 k_4^2\right)}{12\ 2^{2/3} b_1 \sqrt[3]{\beta} d_1 k_4} + \frac{\left(1 + i\sqrt{3}\right)\sqrt[3]{\beta}}{24 \sqrt[3]{2} b_1 d_1 k_4} - \frac{1}{3 b_1}, \tag{A14}$$

where the latter two equilibria are complex conjugates of one another.

The maximum value of $\bar{c}(t)$ can be found by determining by the maximum possible value

of c_{eq}, which occurs when $x = 0$ in $c_{eq}^{(2)}$

$$\max(c_{eq}) = -\frac{1}{6b_1}\left(\frac{2^{2/3}d_1\left(3b_1^2k_2 + 2k_4\right)}{\sqrt[3]{3\sqrt{6}\sqrt{-d_1^6k_2k_4^3\left(b_1^3k_2 - 2b_1k_4\right)^2} + 2d_1^3k_4^2\left(2k_4 - 9b_1^2k_2\right)}}\right. \tag{A15}$$

$$\left. + \frac{\sqrt[3]{6\sqrt{6}\sqrt{-d_1^6k_2k_4^3\left(b_1^3k_2 - 2b_1k_4\right)^2} + 4d_1^3k_4^2\left(2k_4 - 9b_1^2k_2\right)}}{d_1k_4} + 2\right). \tag{A16}$$

For the parameter values used here, $\max(c_{eq}) = \sqrt{2}/2 \approx 0.707107$

The first turning point, x^*, is found by determining the positive value of x at which the two positive equilibria are equal ($c_{eq}^{(2)} = c_{eq}^{(4)}$), namely

$$x^* = \frac{1}{27a_1^2b_1^2k_1^2k_4}\left(2\left(\sqrt{2}\sqrt{a_1^2d_1^2k_1^2k_4\left(3b_1^2k_2 + 2k_4\right)^3} + 2a_1d_1k_1k_4\left(9b_1^2k_2 - 2k_4\right)\right)\right).$$

Inserting this value into either $c_{eq}^{(3)}$ or $c_{eq}^{(4)}$ yields an estimate of c^*. For the parameter values used here, $x^* \approx 0.4495, c^* \approx 0.3565$.

The second turning point, x^{**}, is found by determining the point where the unstable equilibrium $c_{eq}^{(4)}$ first crosses the x axis,

$$x^{**} = \frac{2d_1k_2}{a_1k_1}.$$

Inserting this equation into $c_{eq}^{(3)}$ yields the value of c^{**}, the point to which the equilibrium value of $\bar{c}$ jumps when x reaches x^{**} from above. For the parameter values used here, $x^{**} = 0.192, c^{**} \approx 0.6287$.

C Calculation of Global Lyapunov Exponents

Lyapunov exponents were calculated numerically using the "renormalization" algorithm originally described by Bennettin et al.[1, 2] First, a long trajectory ($T = 40,000$) was generated from an arbitrary initial condition in order to sample a large range of points on the attractor.

Then, N locations on this attractor were randomly chosen, and the Lyapunov exponents were calculated for trajectories originating at each point using the "renormalization" algorithm. The algorithm depends on several parameters: the renormalization time K, the integration time per renormalization T, and the integration timestep dt. The total time sampled to generate a single estimate of the global Lyapunov exponent is given by $K\,T\,dt$.

In order to test for ergodicity, the Lyapunov exponent was calculated for many short runs, as well as for several long runs, in order to reveal any systematic differences in the Lyapunov exponents over different timescales. For the short runs, $K = 20, T = 50, dt = 0.02, N = 500$, resulting in 500 trajectories of length 20. For the long runs, $K = 50, T = 100, dt = 0.02, N = 100$, resulting in 100 trajectories of length 100. Thus for the two sets of trajectories, the total integration time $(N\,K\,T\,dt)$ used to estimate the global Lyapunov spectrum was the same. The resulting Lyapunov spectra are given in Table A. For each set of Lyapunov exponents, the Kaplan-Yorke fractal dimension (D_{KY}) may be directly calculated.

In general, the shorter simulation runs yielded a wide distribution of estimates for the exponents, primarily due to some initial conditions producing trajectories that remain stuck within the "metastable" slow dynamics on the rim of the teacup attractor. While the distribution of each Lyapunov exponent is multimodal for these short integration times, the median of each distribution was clear from $N = 500$ simulation runs. Importantly, despite the spread in values, the signs of the estimates of each of the three Lyapunov exponents were consistent across all simulations, allowing the estimates of Kaplan-Yorke fractal dimension to be compared across different simulations.

For the set of long simulation runs, the estimates of the Lyapunov exponents had a much narrower range of values, despite the smaller number of samples. This convergence of the estimates suggests that ergodicity is present in the chaotic dynamics because the initial conditions were chosen randomly.[3] Moreover, the estimates of the Lyapunov exponents and Kaplan-Yorke fractal dimension generated from the long simulations agree with the estimates generated from the short trajectories, further implying ergodicity.

Table A The spectrum of global Lyapunov exponents for a large set of many short trajectories, and a small set of several long trajectories, all with initial conditions chosen from random points on the strange attractor. The central values are medians and the error ranges are median absolute deviations.

Short Runs (N=500)	Long runs (N=100)
$\lambda_1 = 4 \times 10^{-3} \pm 2 \times 10^{-3}$	$\lambda_1 = 3.1 \times 10^{-3} \pm 6 \times 10^{-4}$
$\lambda_2 = -3 \times 10^{-4} \pm 9 \times 10^{-3}$	$\lambda_2 = -1 \times 10^{-4} \pm 2 \times 10^{-4}$
$\lambda_3 = -0.715 \pm 0.007$	$\lambda_3 = -0.346 \pm 0.002$
$D_{KY} = 2.005 \pm 0.003$	$D_{KY} = 2.009 \pm 0.002$

D Appropriateness of mean trait gradient dynamics

The gradient dynamics model used herein assumes that 1) the predator-prey dynamics depend solely on the mean of the trait distribution 2) the mean trait dynamics depend only on the current mean trait value and no high-order moments of the trait distribution 3) the additive genetic variance (V) remains constant over long timescales. This results in the form of the dynamical equations used throughout the paper,

$$\dot{x}(t) = x(t)\, r(x(t), y(t), \bar{c}(t), c)\Big|_{c\to\bar{c}} \tag{A17}$$

$$\dot{y}(t) = y(t)\left(f(x(t), y(t)) - \tilde{D}(y(t))\right) \tag{A18}$$

$$\dot{\bar{c}}(t) = V\frac{\partial}{\partial c}\left(r(x(t), y(t), \bar{c}(t), c)\right)\Bigg|_{c\to\bar{c}(t)}. \tag{A19}$$

In this section we note some of the underlying assumptions of these equations, and comment on their applicability.

D.1 Underlying assumptions of the mean trait evolution equation

First we consider the accuracy of assuming that the right-hand side of (A19) depends only on the current values of $x(t), y(t), \bar{c}(t)$. Following the derivation originally given by Lande,[4] for an infinitely large population the mean fitness is given by

$$\bar{r} = \int r(c)p(t,c)\; dc \tag{A20}$$

the dependence of each term on time has been suppressed from the notation; $p(c)$ here represents a snapshot distribution of trait values in the prey population at given time,

$$p(c) = \frac{x(c)}{\int x(c)\, dc} \tag{A21}$$

where the denominator represents the total prey population size. Taking the gradient of (A20) and inserting (A21),

$$\begin{aligned} \frac{\partial \bar{r}}{\partial \bar{c}} &= \frac{\partial}{\partial \bar{c}} \left(\int r(c)p(c)\, dc \right) \\ \frac{\partial \bar{r}}{\partial \bar{c}} &= \left(\int \frac{\partial r(c)}{\partial \bar{c}} p(c)\, dc + \int \frac{\partial p(c)}{\partial \bar{c}} r(c)\, dc \right) \end{aligned} \tag{A22}$$

We now assume that the prey trait distribution has the form of a perturbed normal distribution,

$$p(c) = \frac{1}{\sqrt{2\pi V_c}} e^{-\frac{(c-\bar{c})^2}{2V_c}} \left(1 + \frac{\kappa_3}{3! V_c^{3/2}} H_3 \left(\frac{c - \bar{c}}{\sqrt{V_c}} \right) \right)$$

where $H_3(x) = x^3 - 3x$. This form represents a truncated Gram Charlier A series, an expansion of an arbitrary probability distribution in terms of Hermite polynomials. The parameter V_c represents the variance of the prey trait distribution.

Following Lande's original derivation (see Eq. 6 of the referenced article),[4] terms of the form $\int cp(c)r(c)dc - \bar{c}$ in (A22) are proportional $h^2\dot{\bar{c}}$, where h^2 is the narrow-sense heritability. Rearranging (A22) thus results in a dynamical equation for $\dot{\bar{c}}$,

$$\dot{\bar{c}} = V \frac{\partial r(x, y, \bar{c}, c)}{\partial c} \bigg|_{c \to \bar{c}} - V \int_{\infty}^{\infty} \left(\frac{\kappa_3}{2V_c^2} \frac{1}{\sqrt{2\pi V_c}} e^{-\frac{(c-\bar{c})^2}{2V_c}} \left((c - \bar{c})^2 - V_c \right) r(x, y, c, \bar{c}) \right) dc \tag{A23}$$

where the substitution $V = h^2 V_c$ has been performed; V thus represents the only the additive genetic variation (and not total variation) in the population. The first term in this series is derived in Lande's original paper (which assumes κ_3 and all higher cumulants are zero); it is equivalent to the standard gradient dynamics model.[5, 6] In general, the second

"correction" term in this equation (which arises from a departure from Gaussianity in the trait distribution) cannot be solved analytically.

Comment on the assumption of constant additive genetic variance

As noted by previous investigators, even when an observed phenotypic distribution has strongly non-Gaussian form it is often possible to transform it into a distribution with Gaussian form due to the underlying additivity of the random processes that create the trait distribution.[4, 7] By the same token, a Gaussian with a first-order correction term can be used to describe nontrivial distributions that exhibit skew, given an appropriate coordinate transform.[8, 9] Additionally, it has observed that the moments of genetic distributions tend to remain fixed over time, justifying the assumption of holding V_c and κ_3 constant in some cases.

However, even if the trait distribution has non-stationary V_c, the additive genetic variance V may nonetheless remain stationary. Constant additive genetic variance is a common assumption in models in which gene selection is weak compared to selection on phenotypes.[10, 11] The absence of net directional selection in the model presented here ($\bar{c}(t)$ stays bounded between c^* and c^{**}) further ensures that additive genetic variance remains constant. Lande's original derivation[4] further justifies this assumption by noting several studies of the fossil record[12, 13] that led previous investigators to conclude that the additive genetic variance remains nearly constant. This is because there are mechanisms by which a population may maintain a nearly-constant additive genetic variance even if the phenotypic variance (V_c) varies.[14, 15] Examples include cases in which there is a constant degree of environmental heterogeneity, as well as fixed mating preferences and mutation rates among the population that serve to enforced a fixed degree of overall variation even in the absence of strong selection forces.[10, 11, 16, 17] For this reason, even as the prey population evolves, the additive genetic variation (as determined by the realized heritability observed in response to selection) may stay fixed. Because the dynamical equations used here do not specify the dynamics of reproduction or mutation, but rather just the fitness landscape for traits and the mean trait,

the mean trait dynamics model may be most appropriate for populations that have been observed experimentally to maintain nearly-constant heritability values[18–21]

However, for some cases—such as a prey population that fully speciates, directional selection that occurs for extended epochs, or mating and selection that deplete additive genetic variation—-more advanced models of phenotypic evolution have been developed, which relax the assumption of constant genetic variation.[10, 16, 22]

Numerical estimation of the potential contribution of non-Gaussianity in the trait distribution

In order to determine the potential error in the dynamics introduced by neglecting the second term in (A23), the relative size of this term is computed *ex post facto* for simulations of the system generated for the case when $\kappa_3 = 0$ (the case used in the main text). The relative contribution of the correction terms is determined using the ratio of the first and second terms in (A23)

$$err(t) = \frac{\left.\frac{\partial r(x,y,\bar{c},c)}{\partial c}\right|_{c\to\bar{c}}}{\int_{\infty}^{\infty}\left(\frac{\kappa_3}{2V_c^2}\frac{1}{\sqrt{2\pi V_c}}e^{-\frac{(c-\bar{c})^2}{2V_c}}\left((c-\bar{c})^2-V_c\right)r(x,y,c,\bar{c})\right)dc}$$

This quantity should be as small as possible for the gradient dynamics approximation to remain accurate. It has units of $1/\kappa_3$, and thus sets the maximum acceptable κ_3 in the trait distribution. In order to be conservative, V_c may be set to the same value as V in these simulations—this simplifies the computation and overestimates the potential error of neglecting the second term. For $500,000$ randomly-chosen points on the chaotic attractor (determined using times chosen randomly from a long simulation time 4×10^4), the fitness landscape $r(x, y, c, \bar{c})$ is computed using the same method as in Figure 3. This, in addition to the values of $x(t), y(t), \bar{c}(t)$ at each point in the simulation, allows an estimate of $err(t)$ to be calculated at each timepoint using numerical integration over the fitness landscape. For the points searched, $\bar{err} = 0.0025 \pm .0003$ (median $\pm$ median deviation), and the maximum

value of err was 0.1

Next, in order to establish a reference value for κ_3 that may occur in long-timescale, computationally-prohibitive simulations of many individuals, an "upper bound" for κ_3 was computed using known properties of the fitness landscape. Skewness of the trait distribution would be highest during periods of partial disruptive selection, where a group of individuals begins to populate the transient peak that forms at $c = 0$ as well as the second local maximum at $c \approx \sqrt{2}/2$ (see above for a derivation of these values). This case was approximated by modeling the trait distribution during this period as a bimodal Gaussian distribution with peaks lying at $c = 0$ and $c = \sqrt{2}/2$. The widths (σ_1, σ_2) and relative amplitudes (A_1, A_2) of the two peaks of this bimodal distribution are unknown, however for a normalized distribution $A_1 < 1, A_2 < 1$. This bounded four-parameter space was searched randomly for a maximum value subject to these constraints, resulting in an estimate of the upper bound of $\kappa_3 < 0.53$

Together, these suggest that, typically, the additional term in (A23) is not large enough to affect the dynamics $(0.0025 \times 0.53 \ll 1)$. However, in a worst-case scenario on certain part of the chaotic attractor, the second term may have a small effect on the dynamics $(0.1 \times 0.53 = 0.053)$.

Depending on the initial conditions, a distribution resulting from an exhaustive numerical simulation of many individuals may never reach a κ_3 as high as the upper bound presented above. However, if circumstances arise in which the the additional term in (A23) has an overall effect on the long-term dynamics, this effect would likely be to stabilize the dynamics the the system and lead to transient chaos (in which the dynamics eventually exit the chaotic attractor and seek a stable equilibrium or limit cycle). This is because the negative sign of additional term in (A23) dampens the dynamics when the fitness landscape has sharp peaks and valley. Interestingly, a constant fitness landscape $(r(x, y, c, \bar{c}) = r_0(x, y))$ causes the second term in (A23) to equal zero.

D.2 Analysis of the direct dynamics of the full trait distribution

We can further assess the accuracy of the gradient dynamics model by eliminating (A19) and instead writing the predator-prey system in terms of a full integro-differential equation that depends on the full trait distribution. If $x(t, c)$ denotes the density of prey with trait value c, then the mean trait value becomes

$$\bar{c}(t) = \frac{\int x(t, c) c dc}{\int x(c) dc} \tag{A24}$$

where $\int x(c)dc$ represents the total prey density across all trait values. In this case, the system becomes a system of two coupled ordinary differential equations, the first of which depends on the integral (A24)

$$\dot{x}(t, c) = x(t, c)\ r(x(t, c), y(t), \bar{c}(t), c) \tag{A25}$$

$$\dot{y}(t) = y(t) \left(f(x(t, c), y(t)) - \tilde{D}(y(t)) \right) \tag{A26}$$

Full numerical solution of this system of equations is difficult for long periods due to the requirement that the integral term (A24) be evaluated at every timepoint that the numerical integrator computes the numerical derivative constituting the right hand side of (A25). For this reason, small errors in the computation of the mean trait value accumulate quickly, leading the numerical integrator to converge prohibitively slowly to allow direct comparison of numerical solutions to (A17),(A18),(A19) to those of (A25),(A26) over long timescales.

A simple method of determining the accuracy of the gradient dynamics approximation instead relies on the observing that both the integro-differential equation, and the gradient dynamics approximation, cast the predator prey coevolution problem in terms of first-order dynamics. For this reason, comparing the time evolution of the velocity field (as a function of x, y, and $\bar{c}$) under the two formulations can be used to compare how close the dynamics of the two models would be expected to be, even in the absence of full numerical integration

(in which small errors in the velocity fields can accumulate over time). We thus use the following algorithm to determine the effect having a distribution of trait values, $x(t,c)$, has on the dynamics relative to the gradient dynamics approximation:

1. Using the gradient dynamics model (the three-dimensional system (A17),(A18),(A19)), we simulate a long trajectory in the system that resides on the chaotic attractor, $\mathbf{r}_{gd}(t) \equiv (x_{gd}(t), y_{gd}(t), \bar{c}_{gd}(t))$. Because the dynamics of the system are ergodic for long time periods, we assume that a sufficiently long trajectory adequately samples the dynamical space of the system.

2. The long trajectory, $\mathbf{r}_{gd}(t)$ is inserted into (A17),(A18),(A19) in order to generate a time series for the velocity field as a function of time under the gradient dynamics model, $\mathbf{v}_{gd}(t) \equiv (\dot{x}_{gd}(t), \dot{y}_{gd}(t), \bar{c}_{gd}(t))$.

3. At each trajectory point $\mathbf{r}_{gd}(t)$, a distribution of trait values $x_{id}(t,c)$ is defined such that it is centered on the value of the mean trait value returned by the gradient dynamics simulation, $\bar{c}(t)_{gd}$
$$\frac{\int x_{id}(t,c)\, c\; dc}{\int x_{id}(c) dc} = \bar{c}(t)_{gd}$$
The type of distribution $x_{id}(t,c)$ can be chosen freely; here we use a normal distribution, with the standard deviation of the trait distribution σ_c being a parameter to be varied in order to determine the effect of different distribution widths.

4. The time series of distributions $x_{id}(c,t)$ defined in the previous step, as well as $\mathbf{r}_{gd}(t)$, are plugged into (A25),(A26). This produces a time series of prey trait distributions and predator distributions under the integro-differential equation model, $\mathbf{v}_{id}(t) \equiv (x_{id}(t,c), y_{id}(t,c))$

5. At each timepoint, the trait-averaged instantaneous velocity in the x coordinate is calculated using
$$\bar{\dot{x}}_{id} = \frac{\int \dot{x}_{id}(t,c) x(t,c) dc}{\int x_{id}(t,c) dc}$$

Because the predator dynamics depend only on the total prey density ($\int x(t,c)dc$) then the trait-averaged predator density has the simple form $\bar{\dot{y}}_{id}(t) = \dot{y}_{id}(t)$. This produces a time series of trait-averaged velocity values under the integro-differential equation, $\bar{\mathbf{v}}_{id}(t) \equiv (\bar{\dot{x}}_{id}(t), \bar{\dot{y}}_{id}(t))$.

6. The two time series of velocity values under the gradient dynamics ($\mathbf{v}_{gd}(t)$, from step 2) and integro-differential ($\bar{\mathbf{v}}_{id}(t)$, from step 5) models are compared using the average squared error, which penalizes cases in which the instantaneous velocity greatly differs between the two models. A large squared error suggests that the full integro-differential equation would produce different dynamics at many points on the attractor, resulting in qualitatively different dynamics between the two models that would grow larger as the integration time increases.

7. The above sequence of steps is repeated for different values of the trait distribution, σ_c, in order to determine how larger ranges of trait values in the prey population affect the similarity of the gradient dynamics model and the full integro-differential equation system.

Figure C shows the velocity time series generated using the original gradient dynamics (GD) model, $\dot{\bar{c}}_{GD}(t)$, with several velocity time series generated using the full integro-differential (ID) model $\dot{\bar{c}}_{ID}(t)$ for three different values of the variance parameter σ_c^2. When the variance used in the ID simulation (σ_c^2) is either much lower (blue curve) or much higher (magenta curve) than the value V used in the GD model (black curve), the dynamics appear much jerkier than those observed in the GD model. However, when the ID simulation variance approaches the value of V used in the GD model, the dynamics appear similar (green curve). Figure D shows the similarity score between the velocity time series of the gradient dynamics model and the velocity time series of the integro-differential model, computed as a function of the variance parameter used in the ID model. Similarity is calculated as one minus the mean squared difference between the normalized velocity time series for the two

models, with each calculated for a very long trajectory residing on the chaotic attractor. The peak in similarity appears near the point at which the variances of the two models are roughly the same, suggesting general agreement between the two models. More precise peak determination could be achieved with greater computational resources.

E Supplementary figures

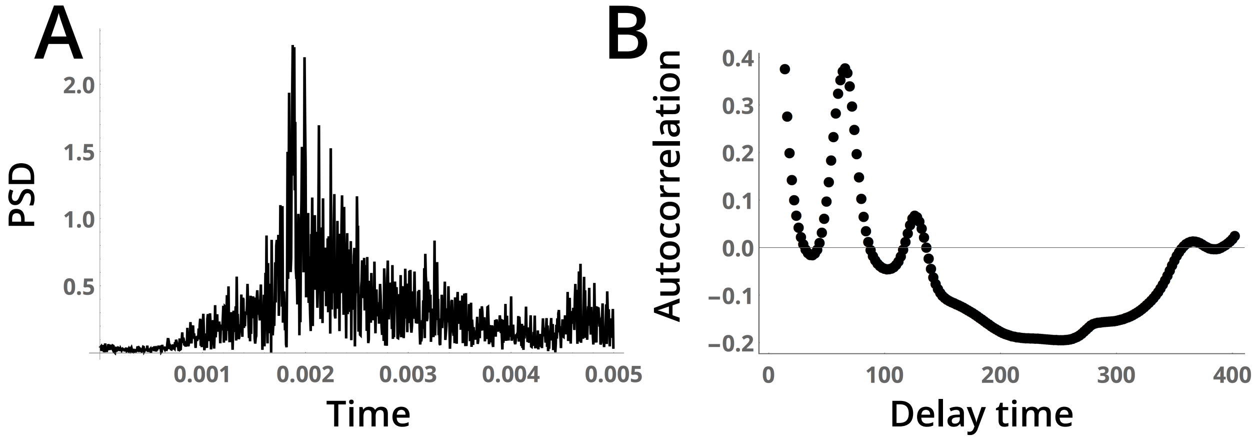

Figure A A. The power spectrum of the chaotic dynamics in $x(t)$. B. The autocorrelation of $x(t)$ as a function of the lag time. The parameter values are the same as those used in Figure 2B.

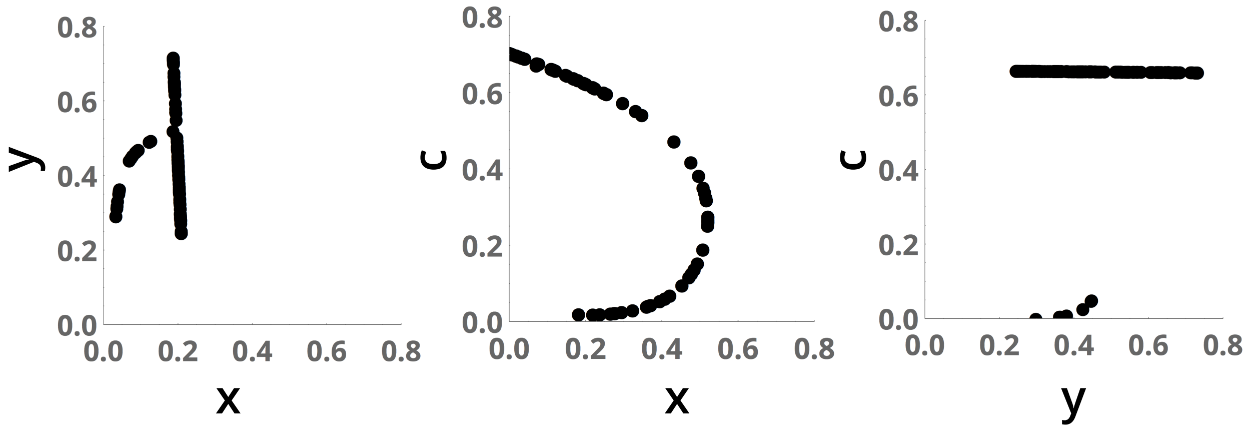

Figure B Poincare sections through the strange attractor, calculated for the same parameter values as used in Figure 2B. For each two-dimensional plot, points are plotted corresponding to the intersection of trajectories with the midplane of the third coordinate. For example, for the (x, y) plot, the $\bar{c}$ midplane is calculated as $c_m ed = (1/T) \int_0^T z dt$. Integration time 1,000,000.

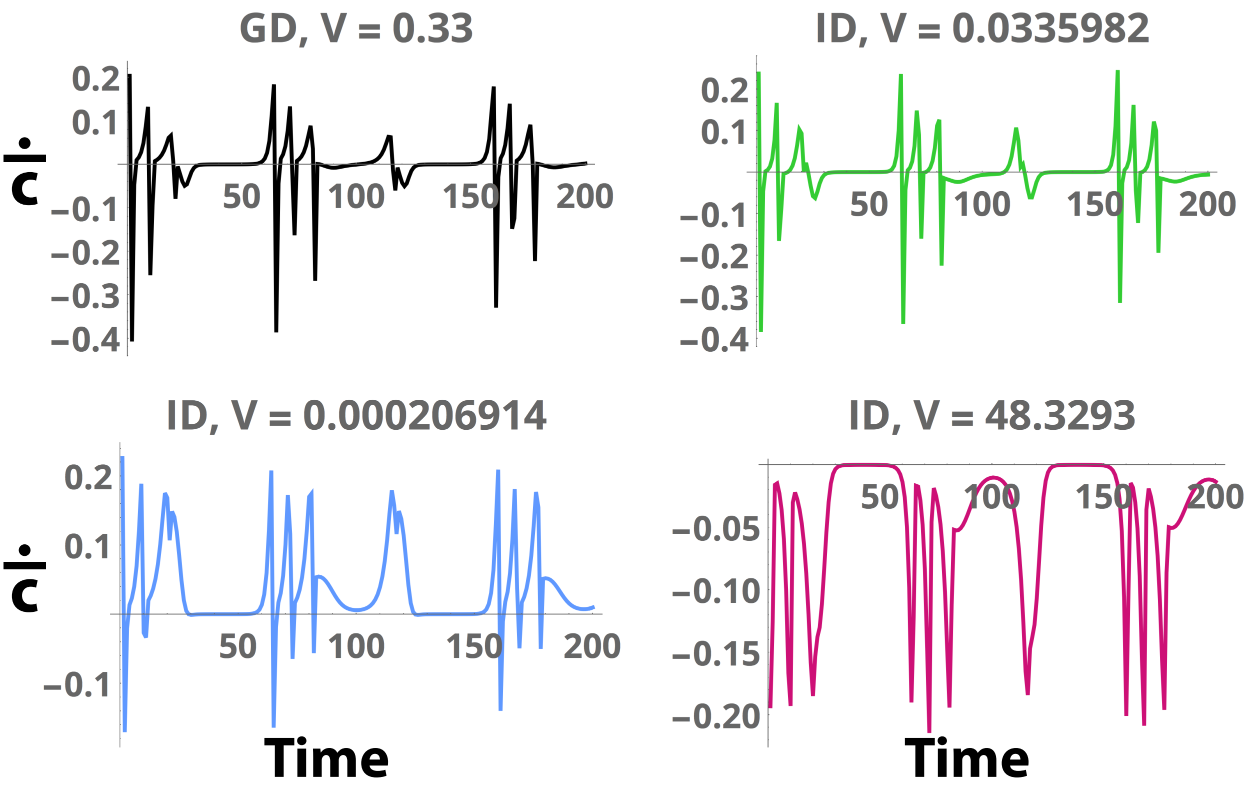

Figure C Comparison of the normalized velocity time series generated by the mean "gradient dynamics" model and the full integro-differential equation evaluated at points along the chaotic attractor. When the variance of the trait distribution used for the integro-differential equation is close to the value used in the mean gradient dynamics simulation, the dynamics appear very similar. All parameters as as given in the main text. Simulation time: 8000.

References

[1] Benettin G, Galgani L, Giorgilli A, Strelcyn JM. Lyapunov characteristic exponents for smooth dynamical systems and for Hamiltonian systems; a method for computing all of them. Part 1: Theory. Meccanica. 1980;15(1):9–20.

[2] Sandri M. Numerical calculation of Lyapunov exponents. Mathematica Journal. 1996;6(3):78–84.

[3] Eckmann JP, Ruelle D. Ergodic theory of chaos and strange attractors. Reviews of modern physics. 1985;57(3):617.

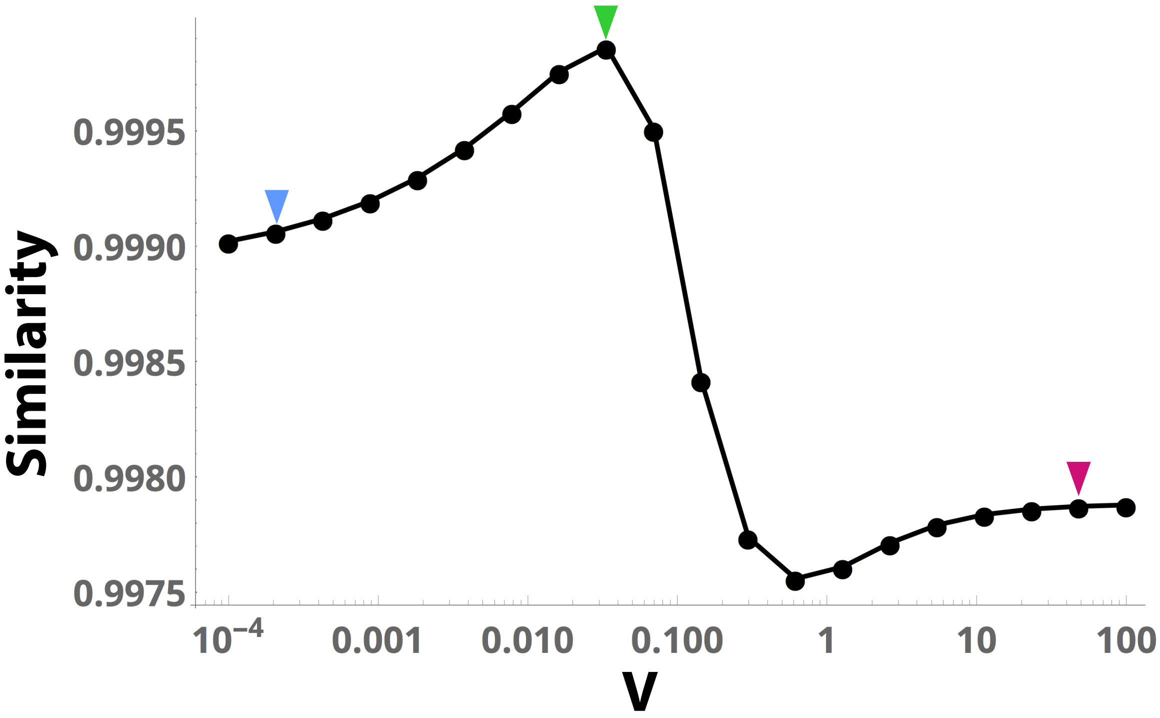

Figure D A comparison of the similarity score (the mean squared error across all time points subtracted from one) between the velocity time series Colored triangles denote the time series shown in Figure C.

[4] Lande R. Natural selection and random genetic drift in phenotypic evolution. Evolution. 1976; p. 314–334.

[5] Cortez MH, Weitz JS. Coevolution can reverse predator–prey cycles. Proceedings of the National Academy of Sciences. 2014;111(20):7486–7491.

[6] Cortez MH. How the Magnitude of Prey Genetic Variation Alters Predator-Prey Eco-Evolutionary Dynamics. The American Naturalist. 2016;188(3):329–341.

[7] Falconer DS. Introduction to quantitative genetics. Pearson Education; 1975.

[8] Nakagawa S, Schielzeth H. Repeatability for Gaussian and non-Gaussian data: a practical guide for biologists. Biological Reviews. 2010;85(4):935–956.

[9] Wright S, et al. Evolution and the genetics of populations. Vol. 1. Genetic and biométrie foundations. Evolution and the genetics of populations Vol 1 Genetic and biométrie foundations. 1968;.

[10] Pennell MW, Harmon LJ. An integrative view of phylogenetic comparative methods: connections to population genetics, community ecology, and paleobiology. Annals of the New York Academy of Sciences. 2013;1289(1):90–105.

[11] Jones AG, Arnold SJ, Burger R. Evolution and stability of the G-matrix on a landscape with a moving optimum. Evolution. 2004;58(8):1639–1654.

[12] Bader RS. Variability and evolutionary rate in the oreodonts. Evolution. 1955; p. 119–140.

[13] Guthrie RD. Variability in characters undergoing rapid evolution, an analysis of Microtus molars. Evolution. 1965; p. 214–233.

[14] Singh RS, Krimbas CB. Evolutionary genetics: from molecules to morphology. vol. 1. Cambridge University Press; 2000.

[15] Riska B, Prout T, Turelli M. Laboratory estimates of heritabilities and genetic correlations in nature. Genetics. 1989;123(4):865–871.

[16] Kruuk LE, Clutton-Brock TH, Slate J, Pemberton JM, Brotherstone S, Guinness FE. Heritability of fitness in a wild mammal population. Proceedings of the National Academy of Sciences. 2000;97(2):698–703.

[17] Jones AG, Arnold SJ, Bürger R, Houle D. Stability of the G-matrix in a population experiencing pleiotropic mutation, stabilizing selection, and genetic drift. Evolution. 2003;57(8):1747–1760.

[18] Brommer JE, Wilson AJ, Gustafsson L. Exploring the genetics of aging in a wild passerine bird. The American Naturalist. 2007;170(4):643–650.

[19] Brown WM, Beck SR, Lange EM, Davis CC, Kay CM, Langefeld CD, et al. Age-stratified heritability estimation in the Framingham Heart Study families. BMC genetics. 2003;4(1):S32.

[20] Kroon J, Andersson B, Mullin TJ. Genetic variation in the diameter–height relationship in Scots pine (Pinus sylvestris). Canadian journal of forest research. 2008;38(6):1493–1503.

[21] Wilson A, Pemberton J, Pilkington J, Clutton-Brock T, Coltman D, Kruuk L. Quantitative genetics of growth and cryptic evolution of body size in an island population. Evolutionary Ecology. 2007;21(3):337–356.

[22] Estes S, Arnold SJ. Resolving the paradox of stasis: models with stabilizing selection explain evolutionary divergence on all timescales. The American Naturalist. 2007;169(2):227–244.
